# Supplementary material for: Tailed giant Tupanvirus possesses the most complete translational apparatus of the known virosphere
Source: Nat Commun. 2018 Feb 27;9:749. doi: 10.1038/s41467-018-03168-1 (PMC5829246; doi:10.1038/s41467-018-03168-1)
Supplement: Supplementary file 1 — Supplementary Information [file 41467_2018_3168_MOESM1_ESM.pdf]

SUPPLEMENTARY INFORMATION

SUPPLEMENTARY NOTE 1

Proteomic analysis of Tupanvirus particles

Proteomic analysis of Tupanvirus soda lake particles revealed 127 proteins, nearly half (32/127 = 25.1%) of which are unknown and eight of which are encoded by ORFans (11/87 = 12.6%). Besides the expected major structural components [major capsid protein (L679) and core protein (L614)], transcription components constitute the largest functional category associated to the viral particle (13 gene products, including subunits of DNA-dependent RNA polymerase, RNA helicases, and mRNA capping enzyme). Moreover, other functional categories were detected in Tupanvirus particles, including protein/lipid modification (14), oxidative pathways (6), DNA topology and repair (3), particle structure (4), and others (87). 59 and 25 out of 127 TPV virion-associated proteins are shared with mimivirus (APMV) and Cafeteria roenbergensis virus respectivelly, out spanning all functional categories analyzed (Supplementary Data 2).

18S rRNA intronic sequence within Mimiviridae and Tupanvirus

The study of 18S rRNA sequences in tupanviruses showed that both copies are located in an intergenic region (Fig. 6; Supplementary Fig. 6A,B). The genomic environment analysis revealed the presence of many hypothetical proteins and ORFans next to the two copies and a tRNA island, composed of seven tRNAs, 15 kbp away from the core sequence of copy 1 (Supplementary Fig. 6A,B). Best hit analysis showed that the first 100 sequences, for both copies, belong mainly to the intronic regions of 18S sequences from different organisms such as fungi, amoebas and algae (Supplementary Fig. 6C,D). The presence of the 18S rRNA intronic sequence was also observed in members of lineages A, B and C of the *Mimiviridae* family (Fig. 6) Lineages A and B presented just

one copy which is located in an intronic region next to a self-splicing group I intron endonuclease. Lineage C presented two copies of the 18S rRNA sequence: copy 1 was disposed in a similar pattern of the copies presented in both tupanviruses (in intergenic regions) and copy 2 showed a most similar pattern to the unique copies of lineages B and C (in an intronic region also next to a self-splicing group I intron endonuclease) (Fig. 6 A-E). Phylogenetic analyses suggested that 18S rRNA copies 1 and 2 of mimiviruses lineage C had separate and different origins. However, Tupanvirus 18S rRNA copy 1 and 2 seem to be related to mimiviruses lineage A and B (single copy) and fungi mitochondrial 18S rRNA intronic region (Fig. 6F). We also observed the presence of three or four copies of the 18S-like sequences in some *Chlorella virus*, which were all phylogenetically inter-related (Fig. 6F). Analyses involving FISH and qPCR of the intronic 18S ribosomal region in tupanvirus soda lake demonstrated that, although these 18S rRNA copies are located in intergenic regions, they are highly expressed during the entire infection, especially in intermediate and later phases (6 and 12 hours post infection) (Supplementay Fig. 8).

Profile of infectiveness of Tupanvirus

Tupanvirus was first isolated in both *Acanthamoeba castellanii* and *Vermamoeba vermiformis,* suggesting a broader host-range compared to other previously described amoebal giant viruses. In light of this, we tested the infectiveness of the new isolate on a large panel of protozoa. Four distinct infectiveness profiles were observed (Supplementary Table 1). Cytopathic effect (CPE), increase of viral titer, and genome replication were observed in *A. castellanii, A. polyphaga, A. sp E4, A. griffini, V. vermiformis, Dysctiostelium discoideum,* and *Willartia magna*, characterizing a productive cycle wherein these hosts were permissive to Tupanvirus. An abortive cycle

was observed in *Acanthamoeba sp michelline* and *A. royreba,* wherein CPE and Tupanvirus genome replication were observed, but there was no particle formation since the viral titer did not increase. *Trichomonas tenax* were completely refractory to Tupanvirus, since CPE, genome replication and increase of viral titer were not observed. The fourth profile was observed in RAW247, THP-1 cells and in *Tetrahymena hyperangularis*, a ravenous free-living protist, wherein Tupanvirus was able to induce a cytopathic effect, but neither an increase of viral titer nor genome replication were observed, thus we concluded that TPV was toxic to the susceptible cell - an unprecedented profile among amoebal giant viruses.

Tupanvirus modulates a non-host organism

Tetrahymena sp was susceptible to Tupanvirus, which caused several effects (Fig. 7G; Fig. 8); however it could not replicate within the protist. In light of this, we hypothesized that the reduction of physiological activity of a (non-host) predator could increase the virus' fitness. We put it to the test by performing *in vitro* simulations, wherein *Acanthamoeba castellanii* (AC) and *Tetrahymena* sp cells were put together and infected at M.O.I. of 10 (Tupanvirus or APMV), and observed over 12 days. Input of fresh medium and permissive host (AC) was done at days four and eight post infection. After the input, Tupanvirus reached higher titers while APMV was extinguished, since the latter was not able to modulate the predator organism, unlike Tupanvirus (Fig. 8N). When we induced a previous ribosomal RNA shutdown in Tetrahymena with geneticin, APMV was able to survive until the last day of observation, similar to tupanvirus. This indicates that viral particles' toxicity confers an advantage in a habitat of intense competition where giant viruses are abundant (e.g. water environment), favoring the encounter between the virus and a permissive host.

Altogether, this data suggest that viral particles can act as active “non-alive” players favoring viral progeny maintenance, in a distinct way of their canonical role of transmitting genetic information. Considering that Tupanvirus is sister group of mimivirus, such modulation mechanism could be an ancient inheritance from an ancestor of *Mimivirus* or *Mimiviridae*. This new mechanism demonstrates that selective pressures over virion content go beyond the metastability properties in early steps of host infection, and can act as unexpected players to protect the rest of the viral progeny against predation.

rRNA shutdown does not seems to be related to ribophagy

The cytotoxic phenotype caused by Tupanvirus in host (*A. castellanii*) and non-host cells (*Tetrahymena*) is a circumstance never previously described and seems to be related to the shutting down of host ribosomal amounts (Fig. 7C; Fig. 8B,C,D,K,L). In a first moment, we believed that the autophagy might be related to Tupanvirus infection. In order to corroborate or not this hypothesis, we developed other several experiments involving the use of acidification inhibitors (chloroquine and bafilomycin A), the observation of acidification of infected cells, as well as the silencing of the gene that encodes an autophagy-related protein, Atg8-2. The treatment of amoebas with chloroquine (a lysosomotropic agent that accumulates inside the acidic parts of the cell, including endosomes and lysosomes, preventing acidification and, consequently, the fusion of endosomes and lysosomes) or bafilomycin A (a vacuolar H+ ATPase inhibitor that acts in the late phase of autophagy) did not prevented the occurrence of the ribosomal shutdown caused by tupanvirus infection, suggesting that this phenomenon is not dependent of the autophagosomes formation, a hallmark of ribophagy (Fig. 7C,D; Supplementary Fig. 9). In order to confirm this first experiment, we also opted to do the

silencing (siRNA) of Atg8-2, that is one of the ubiquitin-like proteins required for autophagosome formation, representing thus a cell marker related to ribophagy. Curiously, the gene silencing also does not prevent viral induced ribosomal shutdown in *Acanthamoeba castellanii,* corroborating the results observed with the inhibitors treatment (Fig. 7C,D). We also checked the pH of cells infected by tupanvirus or mimivirus, in presence or absence of bafilomycin A. Tupanvirus, in contrast to mimivirus, induces a strong acidification of whole amoebal cytoplasm at the time of 9 hours after infection (Fig. 7B; Suppementay Fig.9A,B). In mimivirus-infected amoebas and non-infected amoebas, it was possible visualize only localized acidic vacuoles, but in a scale quite smaller than that observed to tupanvirus infected cells (Fig 7B; Suppementay Fig.9A,B). Interestingly, the intense acidification caused by tupanvirus is not affected by the bafilomicyn A treatment, suggesting that this decrease in pH could be induced by an another acidification mechanisms not related to H+ ATPase pump (Fig. 7B; Suppementay Fig.9A,B). Although tupanvirus induces a remarkable acidification of host cells concomitantly with the ribosomal RNA shutdown, the biological meaning of such acidification and its possible relation to ribosomal shutdown remain to be investigated. As demonstrated in main data, Tupanvirus (inactivated or not) also causes degradation of host (*Acanthamoeba castellanii*) and non-host (*Tetrahymena*) nucleolus/nucleus (Fig. 7F,G), which may contribute to rRNA shutdown, since the nucleolus is important to ribosomal biogenesis. Taken together, these results indicate that this cytotoxic phenotype caused by tupanvirus is a mechanism not-related to the canonical ribophagy/autophagy process.

SUPPLEMENTARY FIGURES

Supplementary Figure 1: Tupanvirus soda lake cycle in *A. castellanii* observed by immunofluorescence. Cells were infected at a multiplicity of infection of 1 and observed at different time points post-infection. In green, viral particles detected by anti-tupan particle antibody produced in mouse; in red, amoeba cytoskeleton. H: hours post-infection. Scale bar, 10 µm.

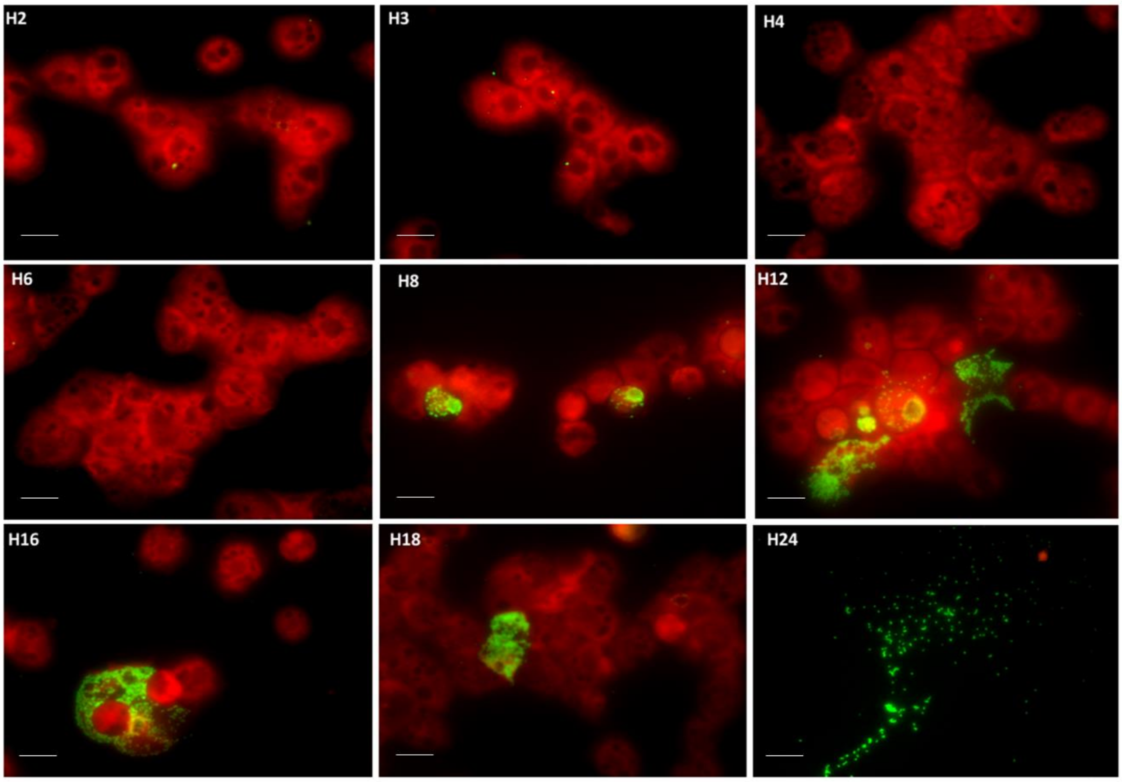

Supplementary Figure 2: Tupanvirus deep ocean genomic information. (a) Rhizome; (b) circular representation of the genome highlighting the translation-related factors; and (c) analysis of Tupanvirus deep ocean 70 tRNAs distribution among amino acid (aa) categories, isoacceptors and their relation to viral aa usage. Bars represent the percentage of use of a given codon (isoacceptor) related to a given aa. Dots above bars represent codons in which Tupanvirus deep ocean presents one or more related tRNAs. *Ambiguous tRNA – also predicted for pyrrolysine.

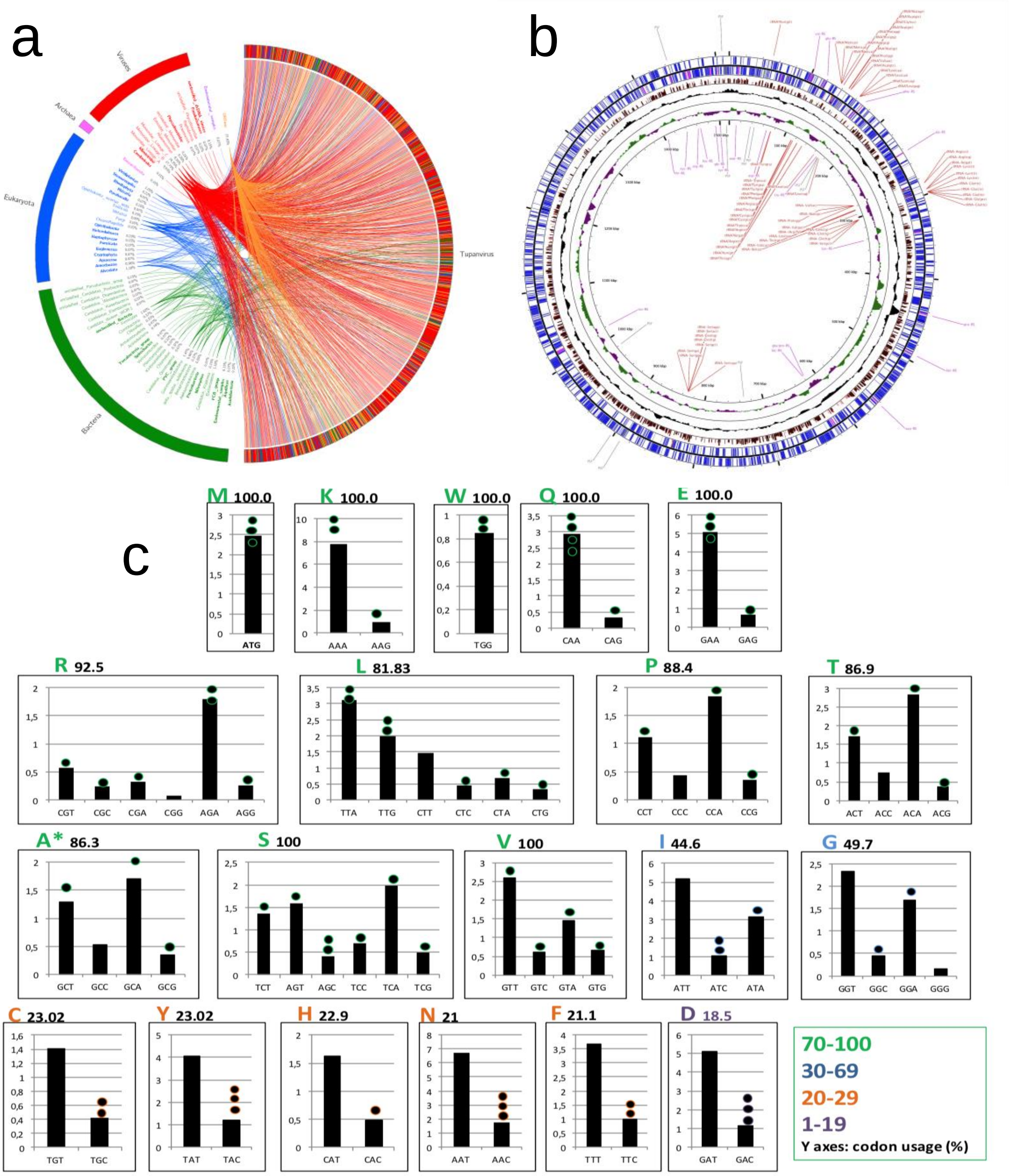

Supplementary Figure 3: Analysis of 67 tRNA distribution in tupanvirus soda lake among amino acid (aa) categories, isoacceptors and their relation to viral aa usage. Bars represent the percentage of use of a given codon (isoacceptor) related to a given aa. Dots above bars represent codons in which Tupanvirus presents one or more related tRNAs. Numbers above the squares represent the percentage of codon occurrence covered by tupanvirus tRNA, considering each aa. These numbers determined the colours, according to the legend at the bottom right corner of the figure.

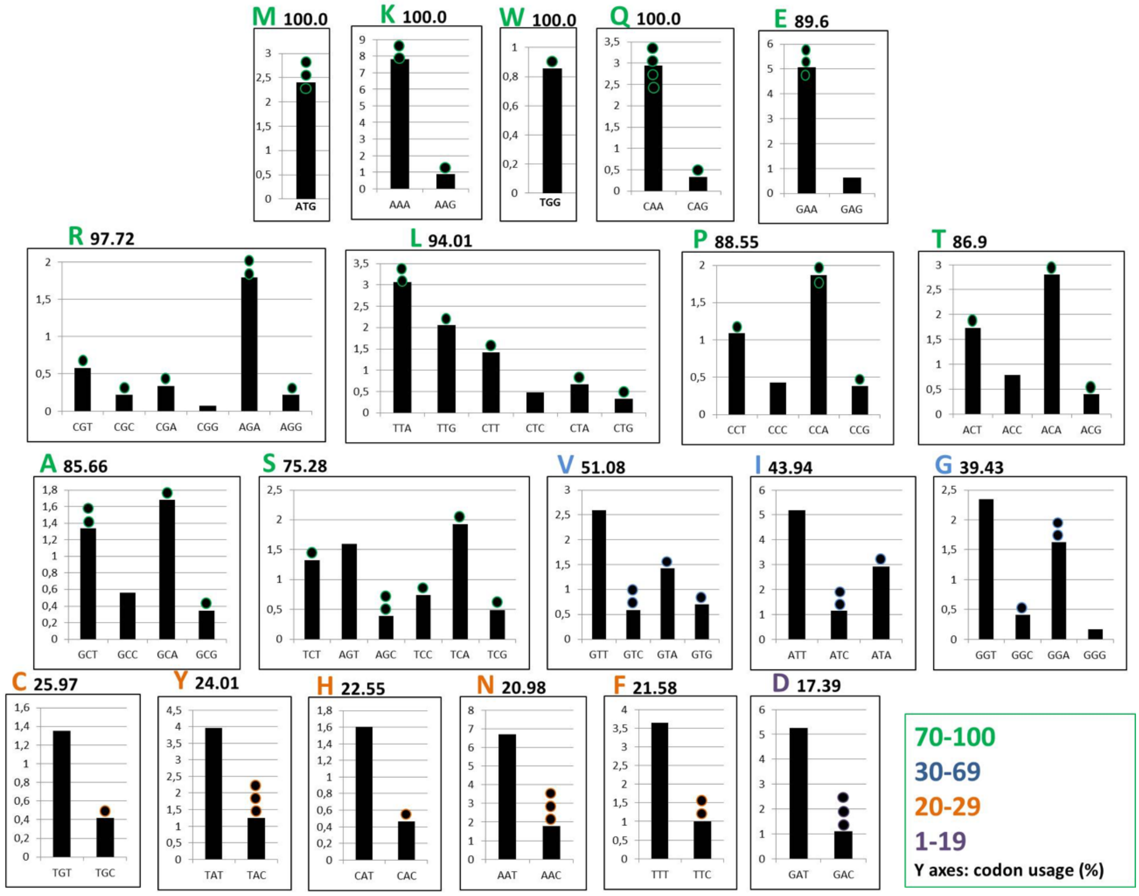

Supplementary Figure 4: Maximum likelihood phylogenetic trees of the 20 Tupanvirus aminoacyl tRNA synthetases (aaRS).

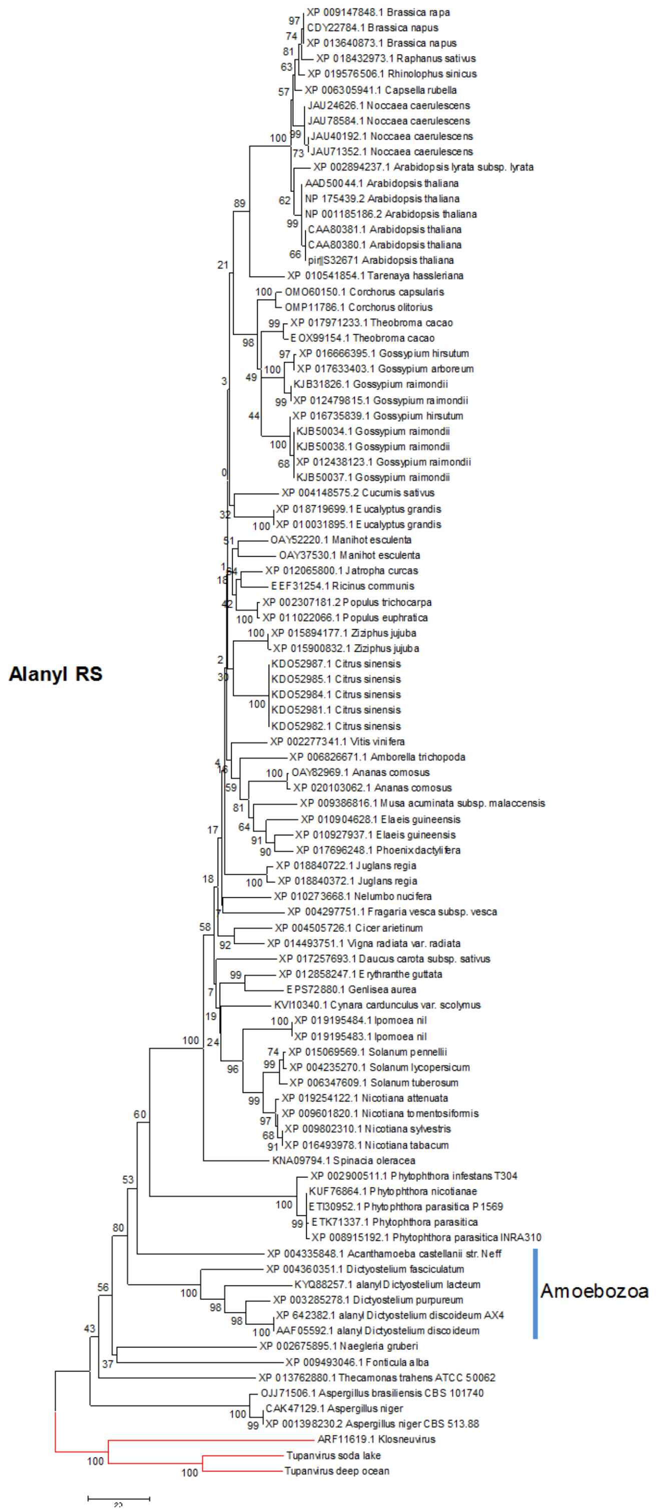

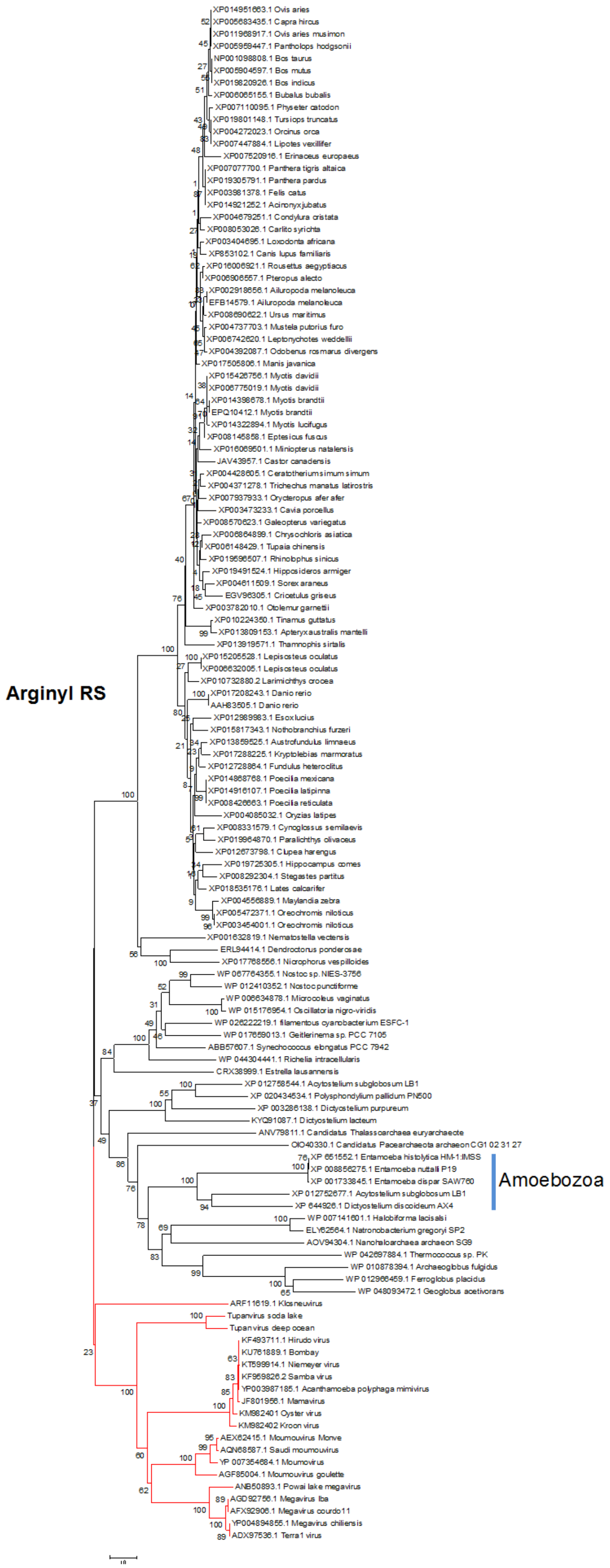

Arginyl RS
XP014951663.1 Ovis aries
XP005683435.1 Capra hircus
XP011968917.1 Ovis aries musimon
XP005959447.1 Pantholops hodgsonii
NP001098808.1 Bos taurus
XP005904597.1 Bos mutus
XP019820926.1 Bos indicus
XP006065155.1 Bubalus bubalis
XP007110095.1 Physeter catodon
XP019801148.1 Tursiops truncatus
XP004272023.1 Orcinus orca
XP007447884.1 Lipotes vexillifer
XP007520916.1 Erinaceus europaeus
XP007077700.1 Panthera tigris altaica
XP019305791.1 Panthera pardus
XP003981378.1 Felis catus
XP014921252.1 Acinonyx jubatus
XP004679251.1 Condylura cristata
XP008053026.1 Carlito syrichta
XP003404695.1 Loxodonta africana
XP853102.1 Canis lupus familiaris
XP016006921.1 Rousettus aegyptiacus
XP006906557.1 Pteropus alecto
XP002918656.1 Ailuropoda melanoleuca
EFB14579.1 Ailuropoda melanoleuca
XP008690622.1 Ursus maritimus
XP004737703.1 Mustela putorius furo
XP006742620.1 Leptonychotes weddellii
XP004392087.1 Odobenus rosmarus divergens
XP017505806.1 Manis javanica
XP015426756.1 Myotis davidii
XP006775019.1 Myotis davidii
XP014398678.1 Myotis brandtii
EPQ10412.1 Myotis brandtii
XP014322894.1 Myotis lucifugus
XP008145858.1 Eptesicus fuscus
XP016069501.1 Miniopterus natalensis
JAV43957.1 Castor canadensis
XP004428605.1 Ceratotherium simum simum
XP004371278.1 Trichechus manatus latirostris
XP007937933.1 Orycteropus afer afer
XP003473233.1 Cavia porcellus
XP008570623.1 Galeopterus variegatus
XP006864899.1 Chrysochloris asiatica
XP006148429.1 Tupaia chinensis
XP019596507.1 Rhinolophus sinicus
XP019491524.1 Hipposideros armiger
XP004611509.1 Sorex araneus
EGV96305.1 Cricetulus griseus
XP003782010.1 Otolemur garnettii
XP010224350.1 Tinamus guttatus
XP013809153.1 Apteryx australis mantelli
XP013919571.1 Thamnophis sirtalis
XP015205528.1 Lepisosteus oculatus
XP006632005.1 Lepisosteus oculatus
XP010732880.2 Larimichthys crocea
XP017208243.1 Danio rerio
AAH83505.1 Danio rerio
XP012989983.1 Esox lucius
XP015817343.1 Nothobranchius furzeri
XP013859525.1 Austrofundulus limnaeus
XP017288225.1 Kryptolebias marmoratus
XP012728864.1 Fundulus heteroclitus
XP014868768.1 Poecilia mexicana
XP014916107.1 Poecilia latipinna
XP008426663.1 Poecilia reticulata
XP004085032.1 Oryzias latipes
XP008331579.1 Cynoglossus semilaevis
XP019964870.1 Paralichthys olivaceus
XP012673798.1 Clupea harengus
XP019725305.1 Hippocampus comes
XP008292304.1 Stegastes partitus
XP018535176.1 Lates calcarifer
XP004556889.1 Maylandia zebra
XP005472371.1 Oreochromis niloticus
XP003454001.1 Oreochromis niloticus
XP001632819.1 Nematostella vectensis
ERL94414.1 Dendroctonus ponderosae
XP017768556.1 Nicrophorus vespilloides
WP 067764355.1 Nostoc sp. NIES-3756
WP 012410352.1 Nostoc punctiforme
WP 006634878.1 Microcoleus vaginatus
WP 015176954.1 Oscillatoria nigro-viridis
WP 026222219.1 filamentous cyanobacterium ESFC-1
WP 017659013.1 Geitlerinema sp. PCC 7105
ABB57607.1 Synechococcus elongatus PCC 7942
WP 044304441.1 Richelia intracellularis
CRX38999.1 Estrella lausannensis
XP 012758544.1 Acytostelium subglobosum LB1
XP 020434534.1 Polysphondylium pallidum PN500
XP 003286138.1 Dictyostelium purpureum
KYQ91087.1 Dictyostelium lacteum
ANV79811.1 Candidatus Thalassoarchaea euryarchaeote
OIO40330.1 Candidatus Pacearchaeota archaeon CG1 02 31 27
XP 651552.1 Entamoeba histolytica HM-1:IMSS
XP 008856275.1 Entamoeba nuttalli P19
XP 001733845.1 Entamoeba dispar SAW760
XP 012752677.1 Acytostelium subglobosum LB1
XP 644926.1 Dictyostelium discoideum AX4
Amoebozoa
WP 007141601.1 Halobiforma lacisalsi
ELY62564.1 Natronobacterium gregoryi SP2
AOV94304.1 Nanohaloarchaea archaeon SG9
WP 042697884.1 Thermococcus sp. PK
WP 010878394.1 Archaeoglobus fulgidus
WP 012966459.1 Ferroglobus placidus
WP 048093472.1 Geoglobus acetivorans
ARF11619.1 Klosneuvirus
Tupanvirus soda lake
Tupanvirus deep ocean
KF493711.1 Hirudo virus
KU761889.1 Bombay
KT599914.1 Niemeyer virus
KF959826.2 Samba virus
YP003987185.1 Acanthamoeba polyphaga mimivirus
JF801956.1 Mamavirus
KM982401 Oyster virus
KM982402 Kroon virus
AEX62415.1 Moumouvirus Monve
AQN68587.1 Saudi moumouvirus
YP 007354684.1 Moumovirus
AGF85004.1 Moumouvirus goulette
ANB50893.1 Powai lake megavirus
AGD92756.1 Megavirus Iba
AFX92906.1 Megavirus courdo11
YP004894855.1 Megavirus chiliensis
ADX97536.1 Terra1 virus

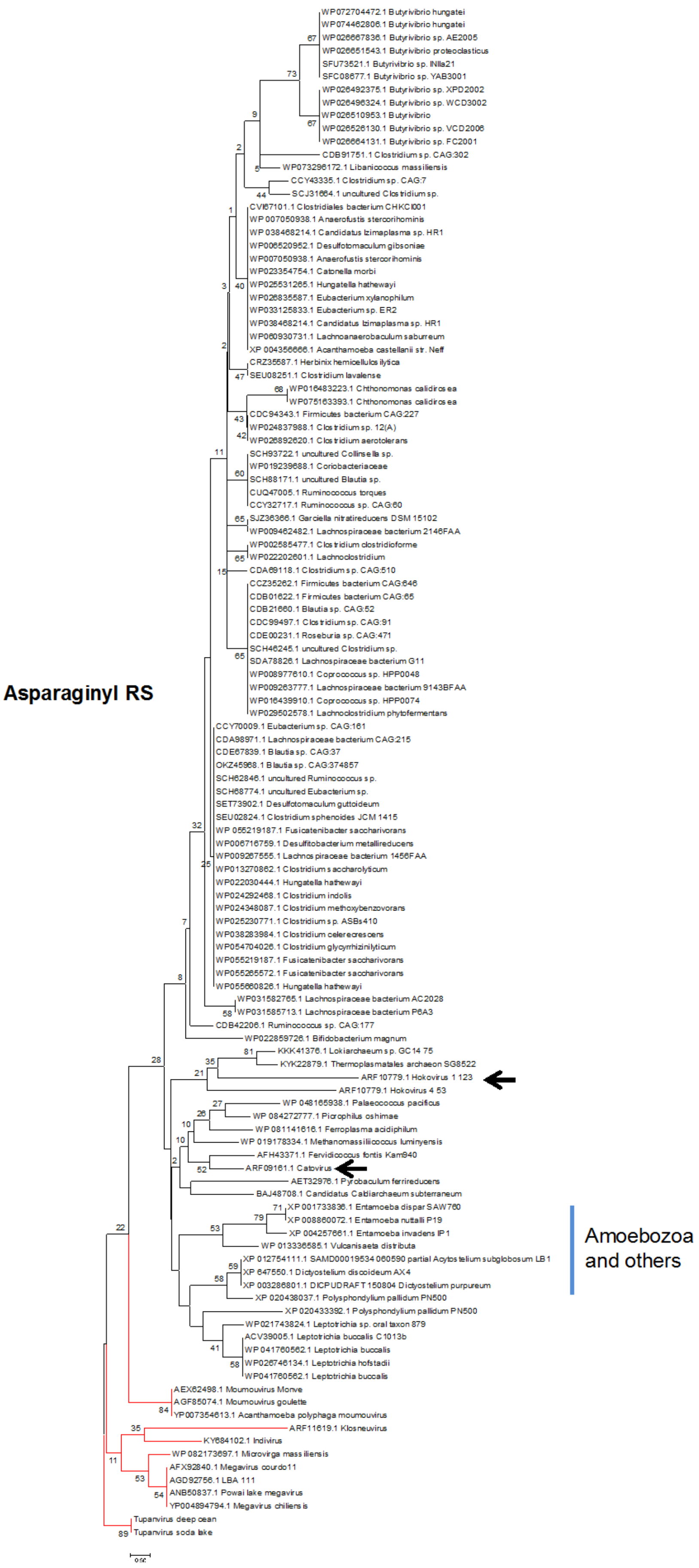

Asparaginyl RS
Amoebozoa and others
WP072704472.1 Butyrivibrio hungatei
WP074462806.1 Butyrivibrio hungatei
WP026667836.1 Butyrivibrio sp. AE2005
WP026651543.1 Butyrivibrio proteoclasticus
SFU73521.1 Butyrivibrio sp. INlla21
SFC08677.1 Butyrivibrio sp. YAB3001
WP026492375.1 Butyrivibrio sp. XPD2002
WP026496324.1 Butyrivibrio sp. WCD3002
WP026510953.1 Butyrivibrio
WP026526130.1 Butyrivibrio sp. VCD2006
WP026664131.1 Butyrivibrio sp. FC2001
CDB91751.1 Clostridium sp. CAG:302
WP073296172.1 Libanicoccus massiliensis
CCY43335.1 Clostridium sp. CAG:7
SCJ31664.1 uncultured Clostridium sp.
CVI67101.1 Clostridiales bacterium CHKCI001
WP 007050938.1 Anaerofustis stercorihominis
WP 038468214.1 Candidatus Izimaplasma sp. HR1
WP006520952.1 Desulfotomaculum gibsoniae
WP007050938.1 Anaerofustis stercorihominis
WP023354754.1 Catonella morbi
WP025531265.1 Hungatella hathewayi
WP026835587.1 Eubacterium xylanophilum
WP033125833.1 Eubacterium sp. ER2
WP038468214.1 Candidatus Izimaplasma sp. HR1
WP060930731.1 Lachnoanaerobaculum saburreum
XP 004356666.1 Acanthamoeba castellanii str. Neff
CRZ35587.1 Herbinix hemicellulosilytica
SEU08251.1 Clostridium lavalense
WP016483223.1 Chthonomonas calidirosea
WP075163393.1 Chthonomonas calidirosea
CDC94343.1 Firmicutes bacterium CAG:227
WP024837988.1 Clostridium sp. 12(A)
WP026892620.1 Clostridium aerotolerans
SCH93722.1 uncultured Collinsella sp.
WP019239688.1 Coriobacteriaceae
SCH88171.1 uncultured Blautia sp.
CUQ47005.1 Ruminococcus torques
CCY32717.1 Ruminococcus sp. CAG:60
SJZ36366.1 Garciella nitratireducens DSM 15102
WP009462482.1 Lachnospiraceae bacterium 2146FAA
WP002585477.1 Clostridium clostridioforme
WP022202601.1 Lachnoclostridium
CDA69118.1 Clostridium sp. CAG:510
CCZ35262.1 Firmicutes bacterium CAG:646
CDB01622.1 Firmicutes bacterium CAG:65
CDB21660.1 Blautia sp. CAG:52
CDC99497.1 Clostridium sp. CAG:91
CDE00231.1 Roseburia sp. CAG:471
SCH46245.1 uncultured Clostridium sp.
SDA78826.1 Lachnospiraceae bacterium G11
WP008977610.1 Coprococcus sp. HPP0048
WP009263777.1 Lachnospiraceae bacterium 9143BFAA
WP016439910.1 Coprococcus sp. HPP0074
WP029502578.1 Lachnoclostridium phytofermentans
CCY70009.1 Eubacterium sp. CAG:161
CDA98971.1 Lachnospiraceae bacterium CAG:215
CDE67839.1 Blautia sp. CAG:37
OKZ45968.1 Blautia sp. CAG:374857
SCH62846.1 uncultured Ruminococcus sp.
SCH68774.1 uncultured Eubacterium sp.
SET73902.1 Desulfotomaculum guttoideum
SEU02824.1 Clostridium sphenoides JCM 1415
WP 055219187.1 Fusicatenibacter saccharivorans
WP006716759.1 Desulfitobacterium metallireducens
WP009267555.1 Lachnospiraceae bacterium 1456FAA
WP013270862.1 Clostridium saccharolyticum
WP022030444.1 Hungatella hathewayi
WP024292468.1 Clostridium indolis
WP024348087.1 Clostridium methoxybenzovorans
WP025230771.1 Clostridium sp. ASBs410
WP038283984.1 Clostridium celerecrescens
WP054704026.1 Clostridium glycyrrhizinilyticum
WP055219187.1 Fusicatenibacter saccharivorans
WP055265572.1 Fusicatenibacter saccharivorans
WP055660826.1 Hungatella hathewayi
WP031582765.1 Lachnospiraceae bacterium AC2028
WP031585713.1 Lachnospiraceae bacterium P6A3
CDB42206.1 Ruminococcus sp. CAG:177
WP022859726.1 Bifidobacterium magnum
KKK41376.1 Lokiarchaeum sp. GC14 75
KYK22879.1 Thermoplasmatales archaeon SG8522
ARF10779.1 Hokovirus 1 123
ARF10779.1 Hokovirus 4 53
WP 048165938.1 Palaeococcus pacificus
WP 084272777.1 Picrophilus oshimae
WP 081141616.1 Ferroplasma acidiphilum
WP 019178334.1 Methanomassiliicoccus luminyensis
AFH43371.1 Fervidicoccus fontis Kam940
ARF09161.1 Catovirus
AET32976.1 Pyrobaculum ferrireducens
BAJ48708.1 Candidatus Caldiarchaeum subterraneum
XP 001733836.1 Entamoeba dispar SAW760
XP 008860072.1 Entamoeba nuttalli P19
XP 004257661.1 Entamoeba invadens IP1
WP 013336585.1 Vulcanisaeta distributa
XP 012754111.1 SAMD00019534 060590 partial Acytostelium subglobosum LB1
XP 647550.1 Dictyostelium discoideum AX4
XP 003286801.1 DICPUDRAFT 150804 Dictyostelium purpureum
XP 020438037.1 Polysphondylium pallidum PN500
XP 020433392.1 Polysphondylium pallidum PN500
WP021743824.1 Leptotrichia sp. oral taxon 879
ACV39005.1 Leptotrichia buccalis C1013b
WP 041760562.1 Leptotrichia buccalis
WP026746134.1 Leptotrichia hofstadii
WP041760562.1 Leptotrichia buccalis
AEX62498.1 Moumouvirus Monve
AGF85074.1 Moumouvirus goulette
YP007354613.1 Acanthamoeba polyphaga moumouvirus
ARF11619.1 Klosneuvirus
KY684102.1 Indivirus
WP 082173697.1 Microvirga massiliensis
AFX92840.1 Megavirus courdo11
AGD92756.1 LBA 111
ANB50837.1 Powai lake megavirus
YP004894794.1 Megavirus chiliensis
Tupanvirus deep ocean
Tupanvirus soda lake

Aspartyl RS

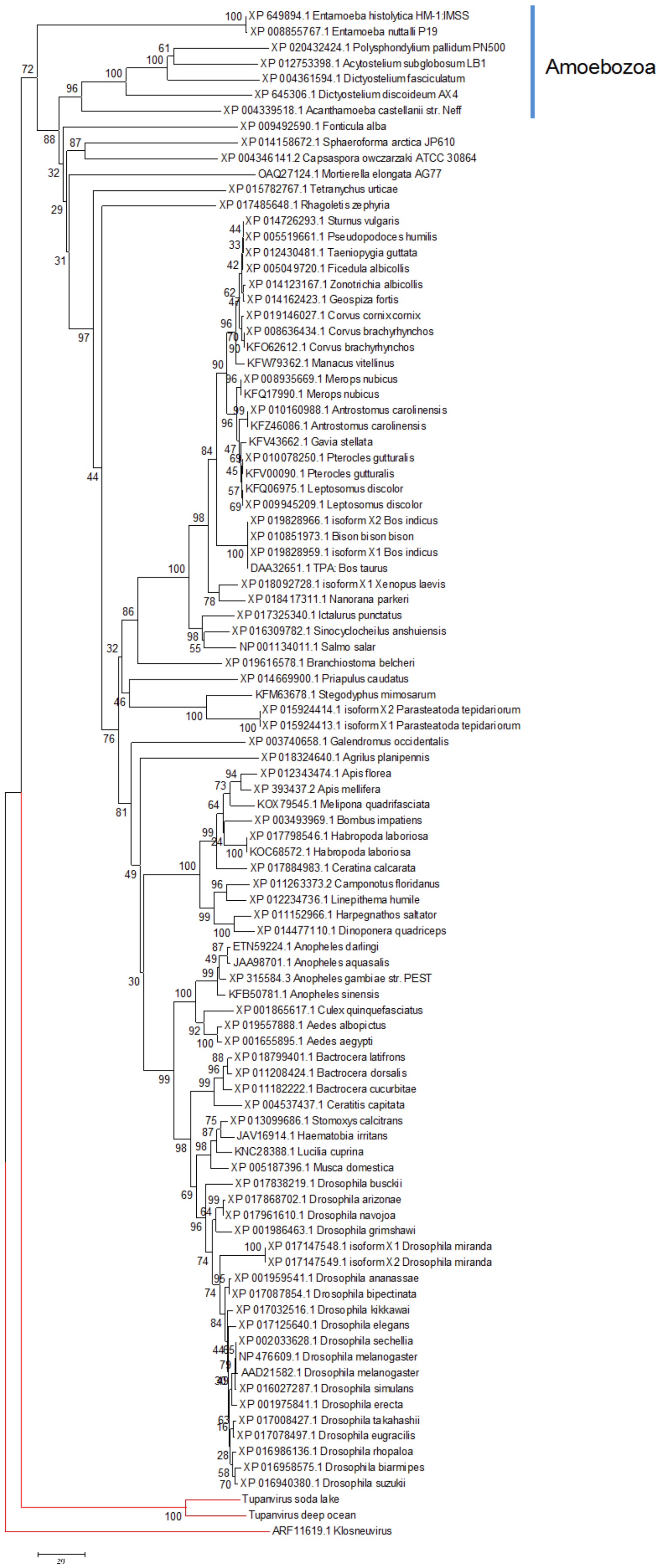
Amoebozoa
XP 649894.1 Entamoeba histolytica HM-1:IMSS
XP 008855767.1 Entamoeba nuttalli P19
XP 020432424.1 Polysphondylium pallidum PN500
XP 012753398.1 Acytostelium subglobosum LB1
XP 004361594.1 Dictyostelium fasciculatum
XP 645306.1 Dictyostelium discoideum AX4
XP 004339518.1 Acanthamoeba castellanii str. Neff
XP 009492590.1 Fonticula alba
XP 014158672.1 Sphaeroforma arctica JP610
XP 004346141.2 Capsaspora owczarzaki ATCC 30864
OAQ27124.1 Mortierella elongata AG77
XP 015782767.1 Tetranychus urticae
XP 017485648.1 Rhagoletis zephyria
XP 014726293.1 Sturnus vulgaris
XP 005519661.1 Pseudopodoces humilis
XP 012430481.1 Taeniopygia guttata
XP 005049720.1 Ficedula albicollis
XP 014123167.1 Zonotrichia albicollis
XP 014162423.1 Geospiza fortis
XP 019146027.1 Corvus cornix cornix
XP 008636434.1 Corvus brachyrhynchos
KFO62612.1 Corvus brachyrhynchos
KFW79362.1 Manacus vitellinus
XP 008935669.1 Merops nubicus
KFQ17990.1 Merops nubicus
XP 010160988.1 Antrostomus carolinensis
KFZ46086.1 Antrostomus carolinensis
KFV43662.1 Gavia stellata
XP 010078250.1 Pterocles gutturalis
KFV00090.1 Pterocles gutturalis
KFQ06975.1 Leptosomus discolor
XP 009945209.1 Leptosomus discolor
XP 019828966.1 isoform X2 Bos indicus
XP 010851973.1 Bison bison bison
XP 019828959.1 isoform X1 Bos indicus
DAA32651.1 TPA: Bos taurus
XP 018092728.1 isoform X1 Xenopus laevis
XP 018417311.1 Nanorana parkeri
XP 017325340.1 Ictalurus punctatus
XP 016309782.1 Sinocyclocheilus anshuiensis
NP 001134011.1 Salmo salar
XP 019616578.1 Branchiostoma belcheri
XP 014669900.1 Priapulus caudatus
KFM63678.1 Stegodyphus mimosarum
XP 015924414.1 isoform X2 Parasteatoda tepidariorum
XP 015924413.1 isoform X1 Parasteatoda tepidariorum
XP 003740658.1 Galendromus occidentalis
XP 018324640.1 Agrilus planipennis
XP 012343474.1 Apis florea
XP 393437.2 Apis mellifera
KOX79545.1 Melipona quadrifasciata
XP 003493969.1 Bombus impatiens
XP 017798546.1 Habropoda laboriosa
KOC68572.1 Habropoda laboriosa
XP 017884983.1 Ceratina calcarata
XP 011263373.2 Camponotus floridanus
XP 012234736.1 Linepithema humile
XP 011152966.1 Harpegnathos saltator
XP 014477110.1 Dinoponera quadriceps
ETN59224.1 Anopheles darlingi
JAA98701.1 Anopheles aquasalis
XP 315584.3 Anopheles gambiae str. PEST
KFB50781.1 Anopheles sinensis
XP 001865617.1 Culex quinquefasciatus
XP 019557888.1 Aedes albopictus
XP 001655895.1 Aedes aegypti
XP 018799401.1 Bactrocera latifrons
XP 011208424.1 Bactrocera dorsalis
XP 011182222.1 Bactrocera cucurbitae
XP 004537437.1 Ceratitis capitata
XP 013099686.1 Stomoxys calcitrans
JAV16914.1 Haematobia irritans
KNC28388.1 Lucilia cuprina
XP 005187396.1 Musca domestica
XP 017838219.1 Drosophila busckii
XP 017868702.1 Drosophila arizonae
XP 017961610.1 Drosophila navojoa
XP 001986463.1 Drosophila grimshawi
XP 017147548.1 isoform X1 Drosophila miranda
XP 017147549.1 isoform X2 Drosophila miranda
XP 001959541.1 Drosophila ananassae
XP 017087854.1 Drosophila bipectinata
XP 017032516.1 Drosophila kikkawai
XP 017125640.1 Drosophila elegans
XP 002033628.1 Drosophila sechellia
NP 476609.1 Drosophila melanogaster
AAD21582.1 Drosophila melanogaster
XP 016027287.1 Drosophila simulans
XP 001975841.1 Drosophila erecta
XP 017008427.1 Drosophila takahashii
XP 017078497.1 Drosophila eugracilis
XP 016986136.1 Drosophila rhopaloa
XP 016958575.1 Drosophila biarmipes
XP 016940380.1 Drosophila suzukii
Tupanvirus soda lake
Tupanvirus deep ocean
ARF11619.1 Klosneuvirus

Cysteinyl RS

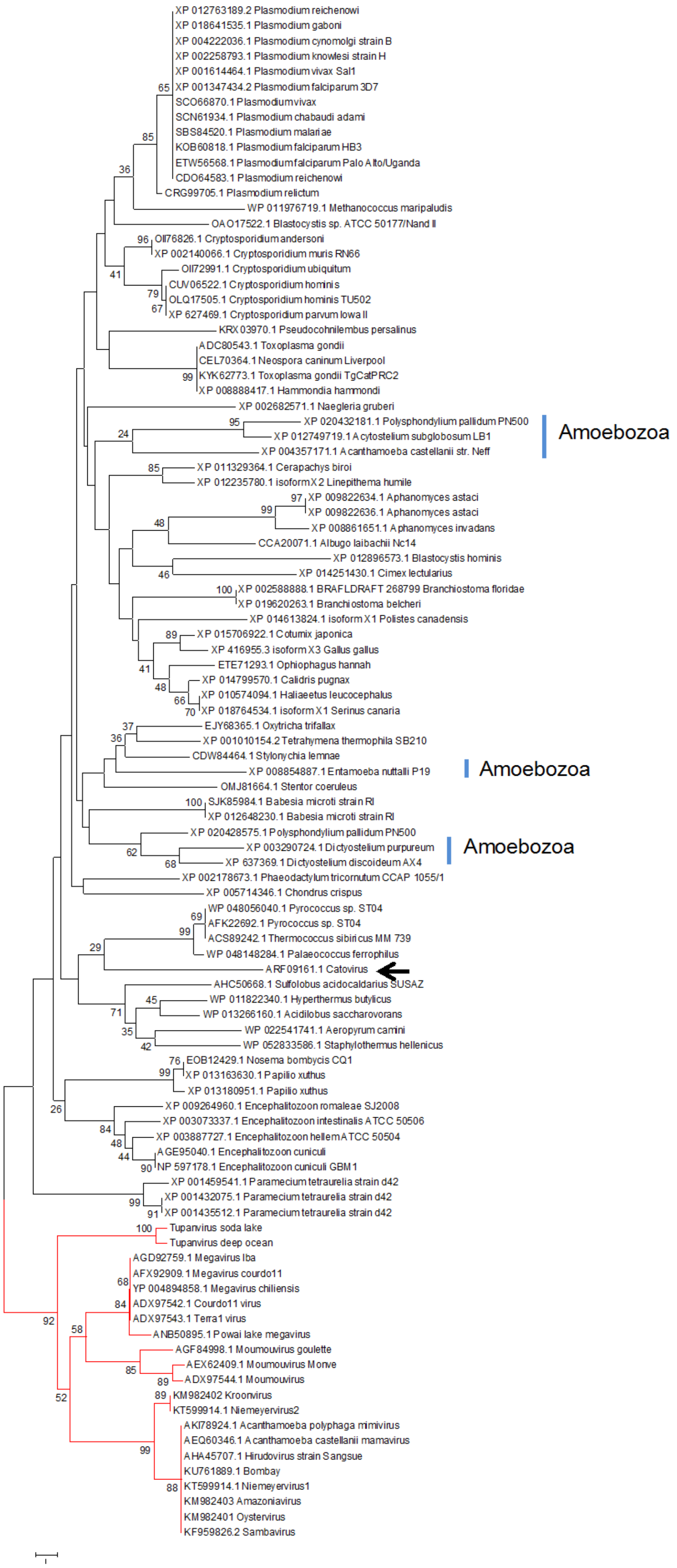

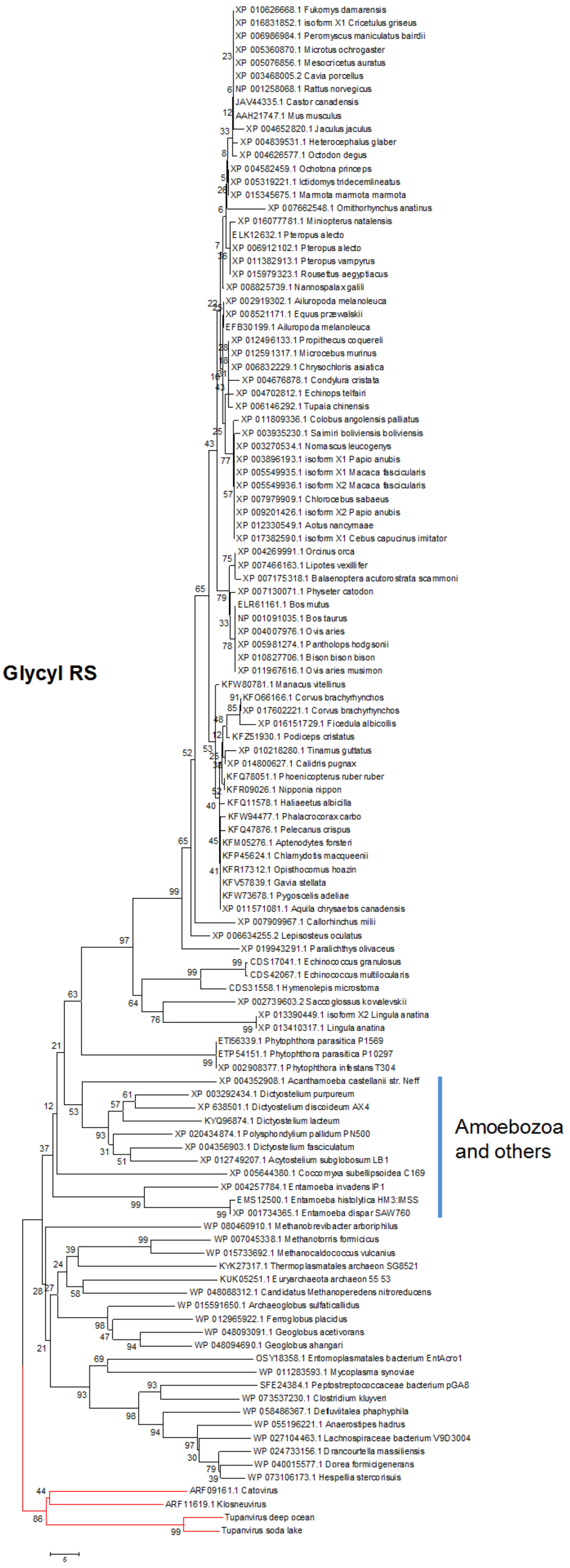
Glycyl RS
Amoebozoa and others
XP 010626668.1 Fukomys damarensis
XP 016831852.1 isoform X1 Cricetulus griseus
XP 006986984.1 Peromyscus maniculatus bairdii
XP 005360870.1 Microtus ochrogaster
XP 005076856.1 Mesocricetus auratus
XP 003468005.2 Cavia porcellus
NP 001258068.1 Rattus norvegicus
JAV44335.1 Castor canadensis
AAH21747.1 Mus musculus
XP 004652820.1 Jaculus jaculus
XP 004839531.1 Heterocephalus glaber
XP 004626577.1 Octodon degus
XP 004582459.1 Ochotona princeps
XP 005319221.1 Ictidomys tridecemlineatus
XP 015345675.1 Marmota marmota marmota
XP 007662548.1 Ornithorhynchus anatinus
XP 016077781.1 Miniopterus natalensis
ELK12632.1 Pteropus alecto
XP 006912102.1 Pteropus alecto
XP 011382913.1 Pteropus vampyrus
XP 015979323.1 Rousettus aegyptiacus
XP 008825739.1 Nannospalax galili
XP 002919302.1 Ailuropoda melanoleuca
XP 008521171.1 Equus przewalskii
EFB30199.1 Ailuropoda melanoleuca
XP 012496133.1 Propithecus coquereli
XP 012591317.1 Microcebus murinus
XP 006832229.1 Chrysochloris asiatica
XP 004676878.1 Condylura cristata
XP 004702812.1 Echinops telfairi
XP 006146292.1 Tupaia chinensis
XP 011809336.1 Colobus angolensis palliatus
XP 003935230.1 Saimiri boliviensis boliviensis
XP 003270534.1 Nomascus leucogenys
XP 003896193.1 isoform X1 Papio anubis
XP 005549935.1 isoform X1 Macaca fascicularis
XP 005549936.1 isoform X2 Macaca fascicularis
XP 007979909.1 Chlorocebus sabaeus
XP 009201426.1 isoform X2 Papio anubis
XP 012330549.1 Aotus nancymaae
XP 017382590.1 isoform X1 Cebus capucinus imitator
XP 004269991.1 Orcinus orca
XP 007466163.1 Lipotes vexillifer
XP 007175318.1 Balaenoptera acutorostrata scammoni
XP 007130071.1 Physeter catodon
ELR61161.1 Bos mutus
NP 001091035.1 Bos taurus
XP 004007976.1 Ovis aries
XP 005981274.1 Pantholops hodgsonii
XP 010827706.1 Bison bison bison
XP 011967616.1 Ovis aries musimon
KFW80781.1 Manacus vitellinus
KFO66166.1 Corvus brachyrhynchos
XP 017602221.1 Corvus brachyrhynchos
XP 016151729.1 Ficedula albicollis
KFZ51930.1 Podiceps cristatus
XP 010218280.1 Tinamus guttatus
XP 014800627.1 Calidris pugnax
KFQ78051.1 Phoenicopterus ruber ruber
KFR09026.1 Nipponia nippon
KFQ11578.1 Haliaeetus albicilla
KFW94477.1 Phalacrocorax carbo
KFQ47876.1 Pelecanus crispus
KFM05276.1 Aptenodytes forsteri
KFP45624.1 Chlamydotis macqueenii
KFR17312.1 Opisthocomus hoazin
KFV57839.1 Gavia stellata
KFW73678.1 Pygoscelis adeliae
XP 011571081.1 Aquila chrysaetos canadensis
XP 007909967.1 Callorhinchus milii
XP 006634255.2 Lepisosteus oculatus
XP 019943291.1 Paralichthys olivaceus
CDS17041.1 Echinococcus granulosus
CDS42067.1 Echinococcus multilocularis
CDS31558.1 Hymenolepis microstoma
XP 002739603.2 Saccoglossus kowalevskii
XP 013390449.1 isoform X2 Lingula anatina
XP 013410317.1 Lingula anatina
ETI56339.1 Phytophthora parasitica P1569
ETP54151.1 Phytophthora parasitica P10297
XP 002908377.1 Phytophthora infestans T30-4
XP 004352908.1 Acanthamoeba castellanii str. Neff
XP 003292434.1 Dictyostelium purpureum
XP 638501.1 Dictyostelium discoideum AX4
KYQ96874.1 Dictyostelium lacteum
XP 020434874.1 Polysphondylium pallidum PN500
XP 004356903.1 Dictyostelium fasciculatum
XP 012749207.1 Acytostelium subglobosum LB1
XP 005644380.1 Coccomyxa subellipsoidea C169
XP 004257784.1 Entamoeba invadens IP1
EMS12500.1 Entamoeba histolytica HM3:IMSS
XP 001734365.1 Entamoeba dispar SAW760
WP 080460910.1 Methanobrevibacter arboriphilus
WP 007045338.1 Methanotorris formicicus
WP 015733692.1 Methanocaldococcus vulcanius
KYK27317.1 Thermoplasmatales archaeon SG8521
KUK05251.1 Euryarchaeota archaeon 55 53
WP 048088312.1 Candidatus Methanoperedens nitroreducens
WP 015591650.1 Archaeoglobus sulfaticallidus
WP 012965922.1 Ferroglobus placidus
WP 048093091.1 Geoglobus acetivorans
WP 048094690.1 Geoglobus ahangari
OSY18358.1 Entomoplasmatales bacterium EntAcro1
WP 011283593.1 Mycoplasma synoviae
SFE24384.1 Peptostreptococcaceae bacterium pGA8
WP 073537230.1 Clostridium kluyveri
WP 058486367.1 Defluviitalea phaphyphila
WP 055196221.1 Anaerostipes hadrus
WP 027104463.1 Lachnospiraceae bacterium V9D3004
WP 024733156.1 Drancourtella massiliensis
WP 040015577.1 Dorea formicigenerans
WP 073106173.1 Hespellia stercorisuis
ARF09161.1 Catovirus
ARF11619.1 Klosneuvirus
Tupanvirus deep ocean
Tupanvirus soda lake
5

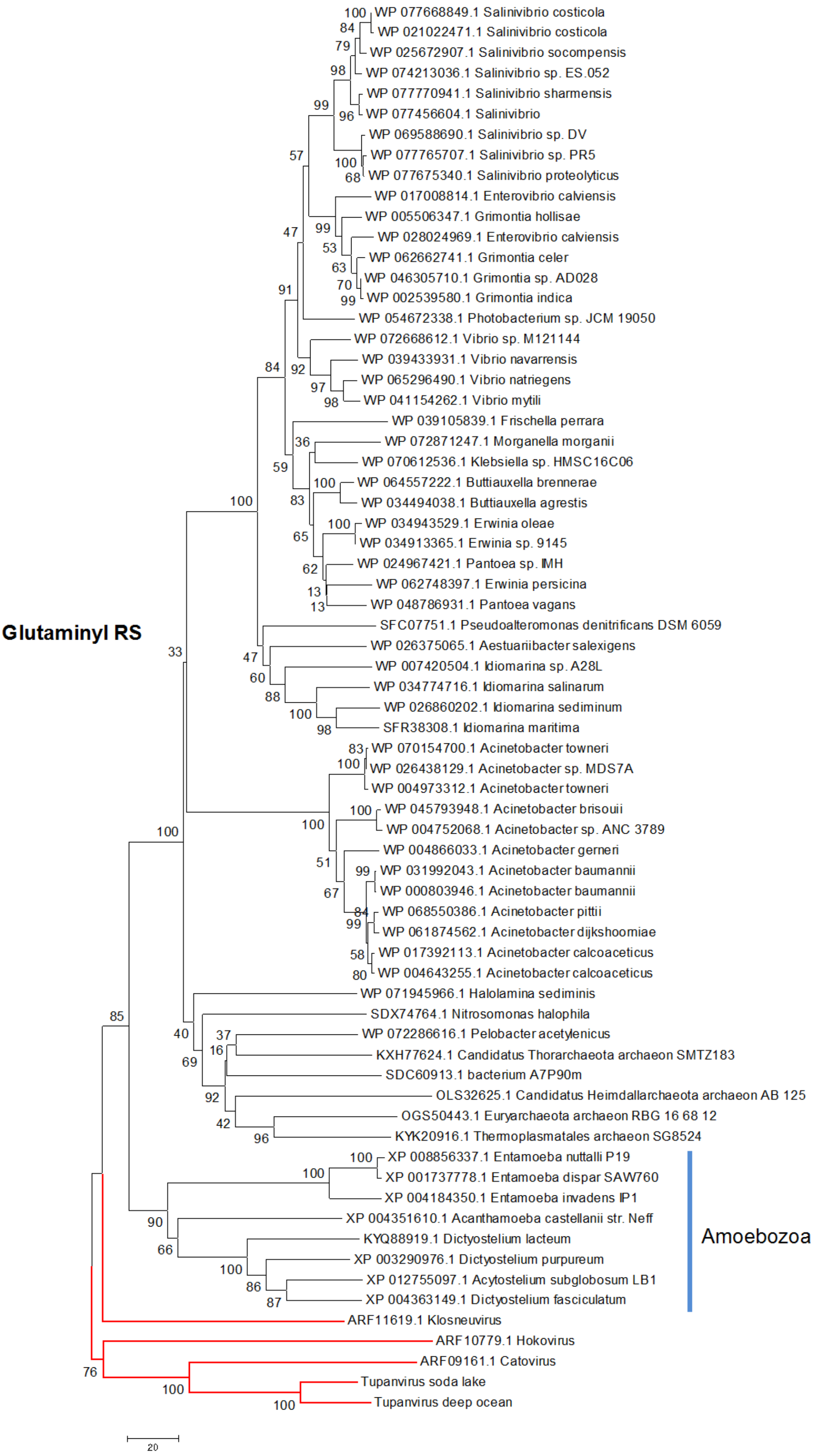

Glutaminyl RS
WP 077668849.1 Salinivibrio costicola
WP 021022471.1 Salinivibrio costicola
WP 025672907.1 Salinivibrio socompensis
WP 074213036.1 Salinivibrio sp. ES.052
WP 077770941.1 Salinivibrio sharmensis
WP 077456604.1 Salinivibrio
WP 069588690.1 Salinivibrio sp. DV
WP 077765707.1 Salinivibrio sp. PR5
WP 077675340.1 Salinivibrio proteolyticus
WP 017008814.1 Enterovibrio calviensis
WP 005506347.1 Grimontia hollisae
WP 028024969.1 Enterovibrio calviensis
WP 062662741.1 Grimontia celer
WP 046305710.1 Grimontia sp. AD028
WP 002539580.1 Grimontia indica
WP 054672338.1 Photobacterium sp. JCM 19050
WP 072668612.1 Vibrio sp. M121144
WP 039433931.1 Vibrio navarrensis
WP 065296490.1 Vibrio natriegens
WP 041154262.1 Vibrio mytili
WP 039105839.1 Frischella perrara
WP 072871247.1 Morganella morganii
WP 070612536.1 Klebsiella sp. HMSC16C06
WP 064557222.1 Buttiauxella brennerae
WP 034494038.1 Buttiauxella agrestis
WP 034943529.1 Erwinia oleae
WP 034913365.1 Erwinia sp. 9145
WP 024967421.1 Pantoea sp. IMH
WP 062748397.1 Erwinia persicina
WP 048786931.1 Pantoea vagans
SFC07751.1 Pseudoalteromonas denitrificans DSM 6059
WP 026375065.1 Aestuariibacter salexigens
WP 007420504.1 Idiomarina sp. A28L
WP 034774716.1 Idiomarina salinarum
WP 026860202.1 Idiomarina sediminum
SFR38308.1 Idiomarina maritima
WP 070154700.1 Acinetobacter towneri
WP 026438129.1 Acinetobacter sp. MDS7A
WP 004973312.1 Acinetobacter towneri
WP 045793948.1 Acinetobacter brisouii
WP 004752068.1 Acinetobacter sp. ANC 3789
WP 004866033.1 Acinetobacter gerneri
WP 031992043.1 Acinetobacter baumannii
WP 000803946.1 Acinetobacter baumannii
WP 068550386.1 Acinetobacter pittii
WP 061874562.1 Acinetobacter dijkshoorniae
WP 017392113.1 Acinetobacter calcoaceticus
WP 004643255.1 Acinetobacter calcoaceticus
WP 071945966.1 Halolamina sediminis
SDX74764.1 Nitrosomonas halophila
WP 072286616.1 Pelobacter acetylenicus
KXH77624.1 Candidatus Thorarchaeota archaeon SMTZ183
SDC60913.1 bacterium A7P90m
OLS32625.1 Candidatus Heimdallarchaeota archaeon AB 125
OGS50443.1 Euryarchaeota archaeon RBG 16 68 12
KYK20916.1 Thermoplasmatales archaeon SG8524
XP 008856337.1 Entamoeba nuttalli P19
XP 001737778.1 Entamoeba dispar SAW760
XP 004184350.1 Entamoeba invadens IP1
XP 004351610.1 Acanthamoeba castellanii str. Neff
KYQ88919.1 Dictyostelium lacteum
XP 003290976.1 Dictyostelium purpureum
XP 012755097.1 Acytostelium subglobosum LB1
XP 004363149.1 Dictyostelium fasciculatum
Amoebozoa
ARF11619.1 Klosneuvirus
ARF10779.1 Hokovirus
ARF09161.1 Catovirus
Tupanvirus soda lake
Tupanvirus deep ocean
20

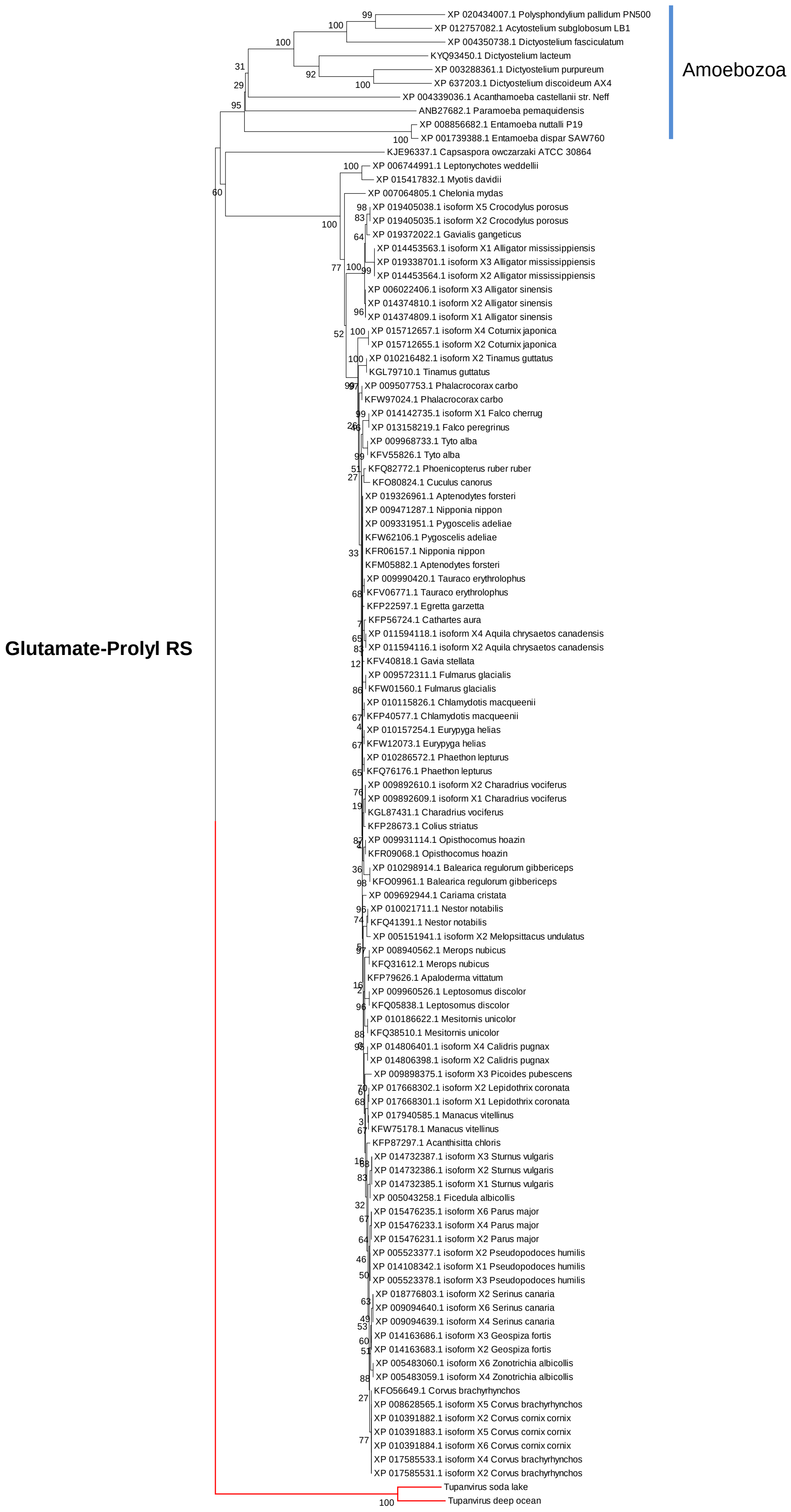
Amoebozoa
Glutamate-Prolyl RS
XP 020434007.1 Polysphondylium pallidum PN500
XP 012757082.1 Acytostelium subglobosum LB1
XP 004350738.1 Dictyostelium fasciculatum
KYQ93450.1 Dictyostelium lacteum
XP 003288361.1 Dictyostelium purpureum
XP 637203.1 Dictyostelium discoideum AX4
XP 004339036.1 Acanthamoeba castellanii str. Neff
ANB27682.1 Paramoeba pemaquidensis
XP 008856682.1 Entamoeba nuttalli P19
XP 001739388.1 Entamoeba dispar SAW760
KJE96337.1 Capsaspora owczarzaki ATCC 30864
XP 006744991.1 Leptonychotes weddellii
XP 015417832.1 Myotis davidii
XP 007064805.1 Chelonia mydas
XP 019405038.1 isoform X5 Crocodylus porosus
XP 019405035.1 isoform X2 Crocodylus porosus
XP 019372022.1 Gavialis gangeticus
XP 014453563.1 isoform X1 Alligator mississippiensis
XP 019338701.1 isoform X3 Alligator mississippiensis
XP 014453564.1 isoform X2 Alligator mississippiensis
XP 006022406.1 isoform X3 Alligator sinensis
XP 014374810.1 isoform X2 Alligator sinensis
XP 014374809.1 isoform X1 Alligator sinensis
XP 015712657.1 isoform X4 Coturnix japonica
XP 015712655.1 isoform X2 Coturnix japonica
XP 010216482.1 isoform X2 Tinamus guttatus
KGL79710.1 Tinamus guttatus
XP 009507753.1 Phalacrocorax carbo
KFW97024.1 Phalacrocorax carbo
XP 014142735.1 isoform X1 Falco cherrug
XP 013158219.1 Falco peregrinus
XP 009968733.1 Tyto alba
KFV55826.1 Tyto alba
KFQ82772.1 Phoenicopterus ruber ruber
KFO80824.1 Cuculus canorus
XP 019326961.1 Aptenodytes forsteri
XP 009471287.1 Nipponia nippon
XP 009331951.1 Pygoscelis adeliae
KFW62106.1 Pygoscelis adeliae
KFR06157.1 Nipponia nippon
KFM05882.1 Aptenodytes forsteri
XP 009990420.1 Tauraco erythrolophus
KFV06771.1 Tauraco erythrolophus
KFP22597.1 Egretta garzetta
KFP56724.1 Cathartes aura
XP 011594118.1 isoform X4 Aquila chrysaetos canadensis
XP 011594116.1 isoform X2 Aquila chrysaetos canadensis
KFV40818.1 Gavia stellata
XP 009572311.1 Fulmarus glacialis
KFW01560.1 Fulmarus glacialis
XP 010115826.1 Chlamydotis macqueenii
KFP40577.1 Chlamydotis macqueenii
XP 010157254.1 Eurypyga helias
KFW12073.1 Eurypyga helias
XP 010286572.1 Phaethon lepturus
KFQ76176.1 Phaethon lepturus
XP 009892610.1 isoform X2 Charadrius vociferus
XP 009892609.1 isoform X1 Charadrius vociferus
KGL87431.1 Charadrius vociferus
KFP28673.1 Colius striatus
XP 009931114.1 Opisthocomus hoazin
KFR09068.1 Opisthocomus hoazin
XP 010298914.1 Balearica regulorum gibbericeps
KFO09961.1 Balearica regulorum gibbericeps
XP 009692944.1 Cariama cristata
XP 010021711.1 Nestor notabilis
KFQ41391.1 Nestor notabilis
XP 005151941.1 isoform X2 Melopsittacus undulatus
XP 008940562.1 Merops nubicus
KFQ31612.1 Merops nubicus
KFP79626.1 Apaloderma vittatum
XP 009960526.1 Leptosomus discolor
KFQ05838.1 Leptosomus discolor
XP 010186622.1 Mesitornis unicolor
KFQ38510.1 Mesitornis unicolor
XP 014806401.1 isoform X4 Calidris pugnax
XP 014806398.1 isoform X2 Calidris pugnax
XP 009898375.1 isoform X3 Picoides pubescens
XP 017668302.1 isoform X2 Lepidothrix coronata
XP 017668301.1 isoform X1 Lepidothrix coronata
XP 017940585.1 Manacus vitellinus
KFW75178.1 Manacus vitellinus
KFP87297.1 Acanthisitta chloris
XP 014732387.1 isoform X3 Sturnus vulgaris
XP 014732386.1 isoform X2 Sturnus vulgaris
XP 014732385.1 isoform X1 Sturnus vulgaris
XP 005043258.1 Ficedula albicollis
XP 015476235.1 isoform X6 Parus major
XP 015476233.1 isoform X4 Parus major
XP 015476231.1 isoform X2 Parus major
XP 005523377.1 isoform X2 Pseudopodoces humilis
XP 014108342.1 isoform X1 Pseudopodoces humilis
XP 005523378.1 isoform X3 Pseudopodoces humilis
XP 018776803.1 isoform X2 Serinus canaria
XP 009094640.1 isoform X6 Serinus canaria
XP 009094639.1 isoform X4 Serinus canaria
XP 014163686.1 isoform X3 Geospiza fortis
XP 014163683.1 isoform X2 Geospiza fortis
XP 005483060.1 isoform X6 Zonotrichia albicollis
XP 005483059.1 isoform X4 Zonotrichia albicollis
KFO56649.1 Corvus brachyrhynchos
XP 008628565.1 isoform X5 Corvus brachyrhynchos
XP 010391882.1 isoform X2 Corvus cornix cornix
XP 010391883.1 isoform X5 Corvus cornix cornix
XP 010391884.1 isoform X6 Corvus cornix cornix
XP 017585533.1 isoform X4 Corvus brachyrhynchos
XP 017585531.1 isoform X2 Corvus brachyrhynchos
Tupanvirus soda lake
Tupanvirus deep ocean

Histidyl RS

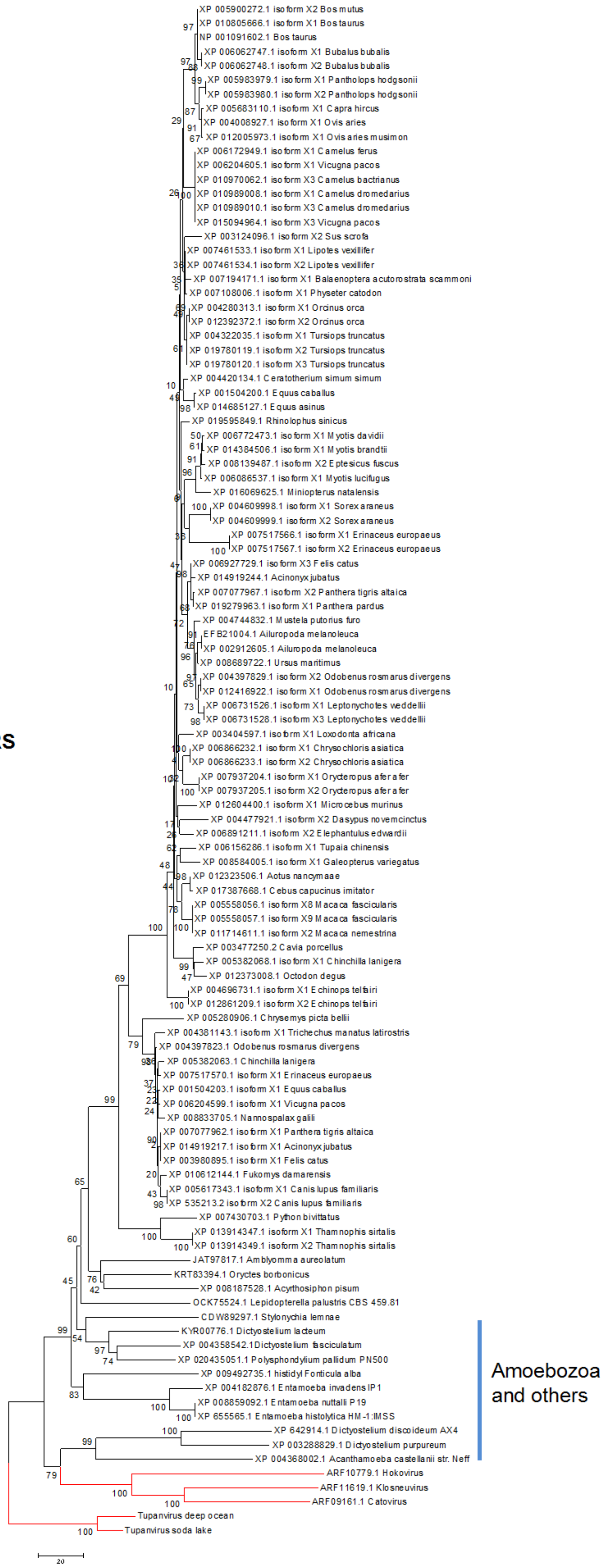
XP 005900272.1 isoform X2 Bos mutus
XP 010805666.1 isoform X1 Bos taurus
NP 001091602.1 Bos taurus
XP 006062747.1 isoform X1 Bubalus bubalis
XP 006062748.1 isoform X2 Bubalus bubalis
XP 005983979.1 isoform X1 Pantholops hodgsonii
XP 005983980.1 isoform X2 Pantholops hodgsonii
XP 005683110.1 isoform X1 Capra hircus
XP 004008927.1 isoform X1 Ovis aries
XP 012005973.1 isoform X1 Ovis aries musimon
XP 006172949.1 isoform X1 Camelus ferus
XP 006204605.1 isoform X1 Vicugna pacos
XP 010970062.1 isoform X3 Camelus bactrianus
XP 010989008.1 isoform X1 Camelus dromedarius
XP 010989010.1 isoform X3 Camelus dromedarius
XP 015094964.1 isoform X3 Vicugna pacos
XP 003124096.1 isoform X2 Sus scrofa
XP 007461533.1 isoform X1 Lipotes vexillifer
XP 007461534.1 isoform X2 Lipotes vexillifer
XP 007194171.1 isoform X1 Balaenoptera acutorostrata scammoni
XP 007108006.1 isoform X1 Physeter catodon
XP 004280313.1 isoform X1 Orcinus orca
XP 012392372.1 isoform X2 Orcinus orca
XP 004322035.1 isoform X1 Tursiops truncatus
XP 019780119.1 isoform X2 Tursiops truncatus
XP 019780120.1 isoform X3 Tursiops truncatus
XP 004420134.1 Ceratotherium simum simum
XP 001504200.1 Equus caballus
XP 014685127.1 Equus asinus
XP 019595849.1 Rhinolophus sinicus
XP 006772473.1 isoform X1 Myotis davidii
XP 014384506.1 isoform X1 Myotis brandtii
XP 008139487.1 isoform X2 Eptesicus fuscus
XP 006086537.1 isoform X1 Myotis lucifugus
XP 016069625.1 Miniopterus natalensis
XP 004609998.1 isoform X1 Sorex araneus
XP 004609999.1 isoform X2 Sorex araneus
XP 007517566.1 isoform X1 Erinaceus europaeus
XP 007517567.1 isoform X2 Erinaceus europaeus
XP 006927729.1 isoform X3 Felis catus
XP 014919244.1 Acinonyx jubatus
XP 007077967.1 isoform X2 Panthera tigris altaica
XP 019279963.1 isoform X1 Panthera pardus
XP 004744832.1 Mustela putorius furo
EFB21004.1 Ailuropoda melanoleuca
XP 002912605.1 Ailuropoda melanoleuca
XP 008689722.1 Ursus maritimus
XP 004397829.1 isoform X2 Odobenus rosmarus divergens
XP 012416922.1 isoform X1 Odobenus rosmarus divergens
XP 006731526.1 isoform X1 Leptonychotes weddellii
XP 006731528.1 isoform X3 Leptonychotes weddellii
XP 003404597.1 isoform X1 Loxodonta africana
XP 006866232.1 isoform X1 Chrysochloris asiatica
XP 006866233.1 isoform X2 Chrysochloris asiatica
XP 007937204.1 isoform X1 Orycteropus afer afer
XP 007937205.1 isoform X2 Orycteropus afer afer
XP 012604400.1 isoform X1 Microcebus murinus
XP 004477921.1 isoform X2 Dasypus novemcinctus
XP 006891211.1 isoform X2 Elephantulus edwardii
XP 006156286.1 isoform X1 Tupaia chinensis
XP 008584005.1 isoform X1 Galeopterus variegatus
XP 012323506.1 Aotus nancymaae
XP 017387668.1 Cebus capucinus imitator
XP 005558056.1 isoform X8 Macaca fascicularis
XP 005558057.1 isoform X9 Macaca fascicularis
XP 011714611.1 isoform X2 Macaca nemestrina
XP 003477250.2 Cavia porcellus
XP 005382068.1 isoform X1 Chinchilla lanigera
XP 012373008.1 Octodon degus
XP 004696731.1 isoform X1 Echinops telfairi
XP 012861209.1 isoform X2 Echinops telfairi
XP 005280906.1 Chrysemys picta bellii
XP 004381143.1 isoform X1 Trichechus manatus latirostris
XP 004397823.1 Odobenus rosmarus divergens
XP 005382063.1 Chinchilla lanigera
XP 007517570.1 isoform X1 Erinaceus europaeus
XP 001504203.1 isoform X1 Equus caballus
XP 006204599.1 isoform X1 Vicugna pacos
XP 008833705.1 Nannospalax galili
XP 007077962.1 isoform X1 Panthera tigris altaica
XP 014919217.1 isoform X1 Acinonyx jubatus
XP 003980895.1 isoform X1 Felis catus
XP 010612144.1 Fukomys damarensis
XP 005617343.1 isoform X1 Canis lupus familiaris
XP 535213.2 isoform X2 Canis lupus familiaris
XP 007430703.1 Python bivittatus
XP 013914347.1 isoform X1 Thamnophis sirtalis
XP 013914349.1 isoform X2 Thamnophis sirtalis
JAT97817.1 Amblyomma aureolatum
KRT83394.1 Oryctes borbonicus
XP 008187528.1 Acyrthosiphon pisum
OCK75524.1 Lepidopterella palustris CBS 459.81
CDW89297.1 Stylonychia lemnae
KYR00776.1 Dictyostelium lacteum
XP 004358542.1 Dictyostelium fasciculatum
XP 020435051.1 Polysphondylium pallidum PN500
XP 009492735.1 histidyl Fonticula alba
XP 004182876.1 Entamoeba invadens IP1
XP 008859092.1 Entamoeba nuttalli P19
XP 655565.1 Entamoeba histolytica HM-1:IMSS
XP 642914.1 Dictyostelium discoideum AX4
XP 003288829.1 Dictyostelium purpureum
XP 004368002.1 Acanthamoeba castellanii str. Neff
ARF10779.1 Hokovirus
ARF11619.1 Klosneuvirus
ARF09161.1 Catovirus
Tupanvirus deep ocean
Tupanvirus soda lake
Amoebozoa and others

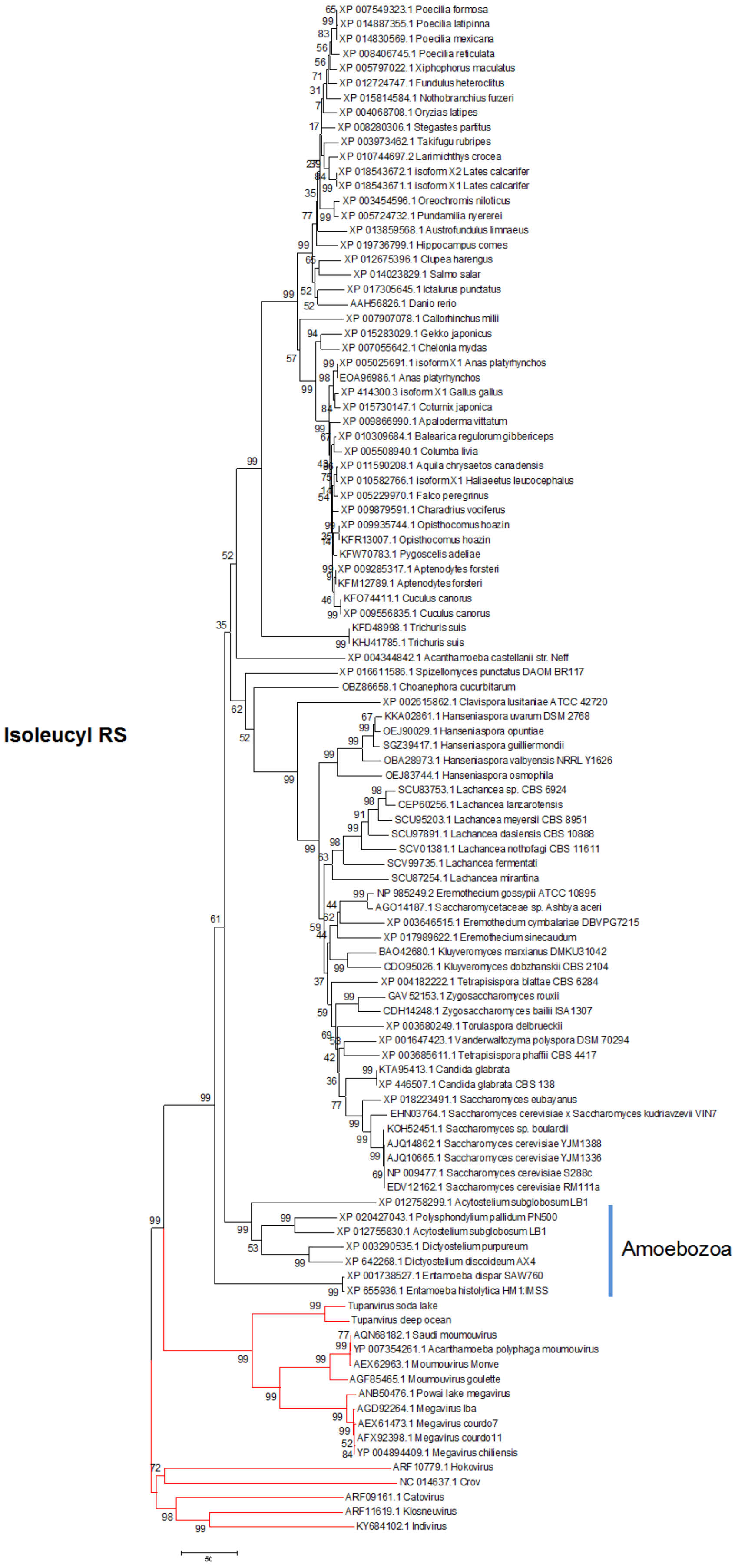
Isoleucyl RS
XP 007549323.1 Poecilia formosa
XP 014887355.1 Poecilia latipinna
XP 014830569.1 Poecilia mexicana
XP 008406745.1 Poecilia reticulata
XP 005797022.1 Xiphophorus maculatus
XP 012724747.1 Fundulus heteroclitus
XP 015814584.1 Nothobranchius furzeri
XP 004068708.1 Oryzias latipes
XP 008280306.1 Stegastes partitus
XP 003973462.1 Takifugu rubripes
XP 010744697.2 Larimichthys crocea
XP 018543672.1 isoform X2 Lates calcarifer
XP 018543671.1 isoform X1 Lates calcarifer
XP 003454596.1 Oreochromis niloticus
XP 005724732.1 Pundamilia nyererei
XP 013859568.1 Austrofundulus limnaeus
XP 019736799.1 Hippocampus comes
XP 012675396.1 Clupea harengus
XP 014023829.1 Salmo salar
XP 017305645.1 Ictalurus punctatus
AAH56826.1 Danio rerio
XP 007907078.1 Callorhinchus milii
XP 015283029.1 Gekko japonicus
XP 007055642.1 Chelonia mydas
XP 005025691.1 isoform X1 Anas platyrhynchos
EOA96986.1 Anas platyrhynchos
XP 414300.3 isoform X1 Gallus gallus
XP 015730147.1 Coturnix japonica
XP 009866990.1 Apaloderma vittatum
XP 010309684.1 Balearica regulorum gibbericeps
XP 005508940.1 Columba livia
XP 011590208.1 Aquila chrysaetos canadensis
XP 010582766.1 isoform X1 Haliaeetus leucocephalus
XP 005229970.1 Falco peregrinus
XP 009879591.1 Charadrius vociferus
XP 009935744.1 Opisthocomus hoazin
KFR13007.1 Opisthocomus hoazin
KFW70783.1 Pygoscelis adeliae
XP 009285317.1 Aptenodytes forsteri
KFM12789.1 Aptenodytes forsteri
KFO74411.1 Cuculus canorus
XP 009556835.1 Cuculus canorus
KFD48998.1 Trichuris suis
KHJ41785.1 Trichuris suis
XP 004344842.1 Acanthamoeba castellanii str. Neff
XP 016611586.1 Spizellomyces punctatus DAOM BR117
OBZ86658.1 Choanephora cucurbitarum
XP 002615862.1 Clavispora lusitaniae ATCC 42720
KKA02861.1 Hanseniaspora uvarum DSM 2768
OEJ90029.1 Hanseniaspora opuntiae
SGZ39417.1 Hanseniaspora guilliermondii
OBA28973.1 Hanseniaspora valbyensis NRRL Y1626
OEJ83744.1 Hanseniaspora osmophila
SCU83753.1 Lachancea sp. CBS 6924
CEP60256.1 Lachancea lanzarotensis
SCU95203.1 Lachancea meyersii CBS 8951
SCU97891.1 Lachancea dasiensis CBS 10888
SCV01381.1 Lachancea nothofagi CBS 11611
SCV99735.1 Lachancea fermentati
SCU87254.1 Lachancea mirantina
NP 985249.2 Eremothecium gossypii ATCC 10895
AGO14187.1 Saccharomycetaceae sp. Ashbya aceri
XP 003646515.1 Eremothecium cymbalariae DBVPG7215
XP 017989622.1 Eremothecium sinecaudum
BAO42680.1 Kluyveromyces marxianus DMKU31042
CDO95026.1 Kluyveromyces dobzhanskii CBS 2104
XP 004182222.1 Tetrapisispora blattae CBS 6284
GAV52153.1 Zygosaccharomyces rouxii
CDH14248.1 Zygosaccharomyces bailii ISA1307
XP 003680249.1 Torulaspora delbrueckii
XP 001647423.1 Vanderwaltozyma polyspora DSM 70294
XP 003685611.1 Tetrapisispora phaffii CBS 4417
KTA95413.1 Candida glabrata
XP 446507.1 Candida glabrata CBS 138
XP 018223491.1 Saccharomyces eubayanus
EHN03764.1 Saccharomyces cerevisiae x Saccharomyces kudriavzevii VIN7
KOH52451.1 Saccharomyces sp. boulardii
AJQ14862.1 Saccharomyces cerevisiae YJM1388
AJQ10665.1 Saccharomyces cerevisiae YJM1336
NP 009477.1 Saccharomyces cerevisiae S288c
EDV12162.1 Saccharomyces cerevisiae RM11-1a
XP 012758299.1 Acytostelium subglobosum LB1
XP 020427043.1 Polysphondylium pallidum PN500
XP 012755830.1 Acytostelium subglobosum LB1
XP 003290535.1 Dictyostelium purpureum
XP 642268.1 Dictyostelium discoideum AX4
XP 001738527.1 Entamoeba dispar SAW760
XP 655936.1 Entamoeba histolytica HM-1:IMSS
Amoebozoa
Tupanvirus soda lake
Tupanvirus deep ocean
AQN68182.1 Saudi moumouvirus
YP 007354261.1 Acanthamoeba polyphaga moumouvirus
AEX62963.1 Moumouvirus Monve
AGF85465.1 Moumouvirus goulette
ANB50476.1 Powai lake megavirus
AGD92264.1 Megavirus lba
AEX61473.1 Megavirus courdo7
AFX92398.1 Megavirus courdo11
YP 004894409.1 Megavirus chiliensis
ARF10779.1 Hokovirus
NC 014637.1 Crov
ARF09161.1 Catovirus
ARF11619.1 Klosneuvirus
KY684102.1 Indivirus

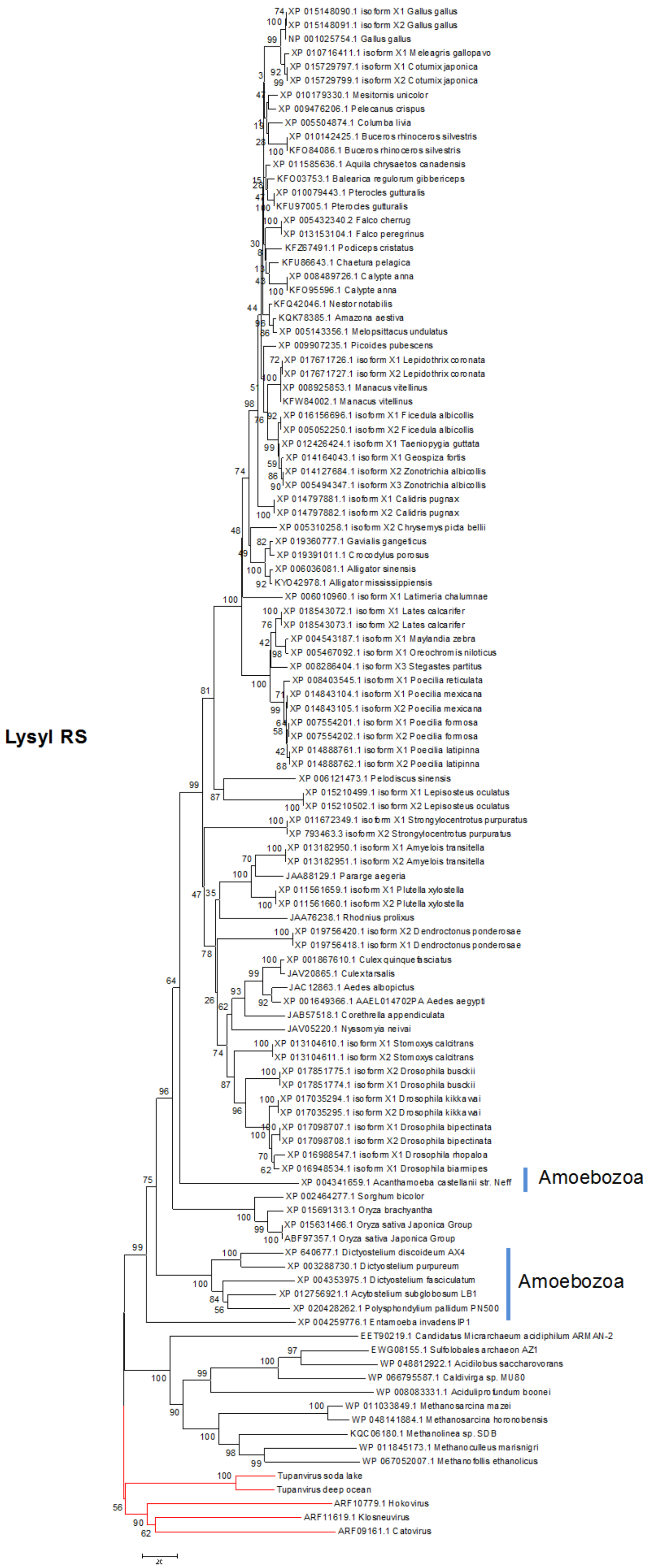
Lysyl RS
XP 015148090.1 isoform X1 Gallus gallus
XP 015148091.1 isoform X2 Gallus gallus
NP 001025754.1 Gallus gallus
XP 010716411.1 isoform X1 Meleagris gallopavo
XP 015729797.1 isoform X1 Coturnix japonica
XP 015729799.1 isoform X2 Coturnix japonica
XP 010179330.1 Mesitornis unicolor
XP 009476206.1 Pelecanus crispus
XP 005504874.1 Columba livia
XP 010142425.1 Buceros rhinoceros silvestris
KFO84086.1 Buceros rhinoceros silvestris
XP 011585636.1 Aquila chrysaetos canadensis
KFO03753.1 Balearica regulorum gibbericeps
XP 010079443.1 Pterocles gutturalis
KFU97005.1 Pterocles gutturalis
XP 005432340.2 Falco cherrug
XP 013153104.1 Falco peregrinus
KFZ67491.1 Podiceps cristatus
KFU86643.1 Chaetura pelagica
XP 008489726.1 Calypte anna
KFO95596.1 Calypte anna
KFQ42046.1 Nestor notabilis
KQK78385.1 Amazona aestiva
XP 005143356.1 Melopsittacus undulatus
XP 009907235.1 Picoides pubescens
XP 017671726.1 isoform X1 Lepidothrix coronata
XP 017671727.1 isoform X2 Lepidothrix coronata
XP 008925853.1 Manacus vitellinus
KFW84002.1 Manacus vitellinus
XP 016156696.1 isoform X1 Ficedula albicollis
XP 005052250.1 isoform X2 Ficedula albicollis
XP 012426424.1 isoform X1 Taeniopygia guttata
XP 014164043.1 isoform X1 Geospiza fortis
XP 014127684.1 isoform X2 Zonotrichia albicollis
XP 005494347.1 isoform X3 Zonotrichia albicollis
XP 014797881.1 isoform X1 Calidris pugnax
XP 014797882.1 isoform X2 Calidris pugnax
XP 005310258.1 isoform X2 Chrysemys picta bellii
XP 019360777.1 Gavialis gangeticus
XP 019391011.1 Crocodylus porosus
XP 006036081.1 Alligator sinensis
KYO42978.1 Alligator mississippiensis
XP 006010960.1 isoform X1 Latimeria chalumnae
XP 018543072.1 isoform X1 Lates calcarifer
XP 018543073.1 isoform X2 Lates calcarifer
XP 004543187.1 isoform X1 Maylandia zebra
XP 005467092.1 isoform X1 Oreochromis niloticus
XP 008286404.1 isoform X3 Stegastes partitus
XP 008403545.1 isoform X1 Poecilia reticulata
XP 014843104.1 isoform X1 Poecilia mexicana
XP 014843105.1 isoform X2 Poecilia mexicana
XP 007554201.1 isoform X1 Poecilia formosa
XP 007554202.1 isoform X2 Poecilia formosa
XP 014888761.1 isoform X1 Poecilia latipinna
XP 014888762.1 isoform X2 Poecilia latipinna
XP 006121473.1 Pelodiscus sinensis
XP 015210499.1 isoform X1 Lepisosteus oculatus
XP 015210502.1 isoform X2 Lepisosteus oculatus
XP 011672349.1 isoform X1 Strongylocentrotus purpuratus
XP 793463.3 isoform X2 Strongylocentrotus purpuratus
XP 013182950.1 isoform X1 Amyelois transitella
XP 013182951.1 isoform X2 Amyelois transitella
JAA88129.1 Pararge aegeria
XP 011561659.1 isoform X1 Plutella xylostella
XP 011561660.1 isoform X2 Plutella xylostella
JAA76238.1 Rhodnius prolixus
XP 019756420.1 isoform X2 Dendroctonus ponderosae
XP 019756418.1 isoform X1 Dendroctonus ponderosae
XP 001867610.1 Culex quinquefasciatus
JAV20865.1 Culex tarsalis
JAC12863.1 Aedes albopictus
XP 001649366.1 AAEL014702PA Aedes aegypti
JAB57518.1 Corethrella appendiculata
JAV05220.1 Nyssomyia neivai
XP 013104610.1 isoform X1 Stomoxys calcitrans
XP 013104611.1 isoform X2 Stomoxys calcitrans
XP 017851775.1 isoform X2 Drosophila busckii
XP 017851774.1 isoform X1 Drosophila busckii
XP 017035294.1 isoform X1 Drosophila kikkawai
XP 017035295.1 isoform X2 Drosophila kikkawai
XP 017098707.1 isoform X1 Drosophila bipectinata
XP 017098708.1 isoform X2 Drosophila bipectinata
XP 016988547.1 isoform X1 Drosophila rhopaloa
XP 016948534.1 isoform X1 Drosophila biarmipes
XP 004341659.1 Acanthamoeba castellanii str. Neff
Amoebozoa
XP 002464277.1 Sorghum bicolor
XP 015691313.1 Oryza brachyantha
XP 015631466.1 Oryza sativa Japonica Group
ABF97357.1 Oryza sativa Japonica Group
XP 640677.1 Dictyostelium discoideum AX4
XP 003288730.1 Dictyostelium purpureum
XP 004353975.1 Dictyostelium fasciculatum
XP 012756921.1 Acytostelium subglobosum LB1
XP 020428262.1 Polysphondylium pallidum PN500
Amoebozoa
XP 004259776.1 Entamoeba invadens IP1
EET90219.1 Candidatus Micrarchaeum acidiphilum ARMAN-2
EWG08155.1 Sulfolobales archaeon AZ1
WP 048812922.1 Acidilobus saccharovorans
WP 066795587.1 Caldivirga sp. MU80
WP 008083331.1 Aciduliprofundum boonei
WP 011033849.1 Methanosarcina mazei
WP 048141884.1 Methanosarcina horonobensis
KQC06180.1 Methanolinea sp. SDB
WP 011845173.1 Methanoculleus marisnigri
WP 067052007.1 Methanofollis ethanolicus
Tupanvirus soda lake
Tupanvirus deep ocean
ARF10779.1 Hokovirus
ARF11619.1 Klosneuvirus
ARF09161.1 Catovirus

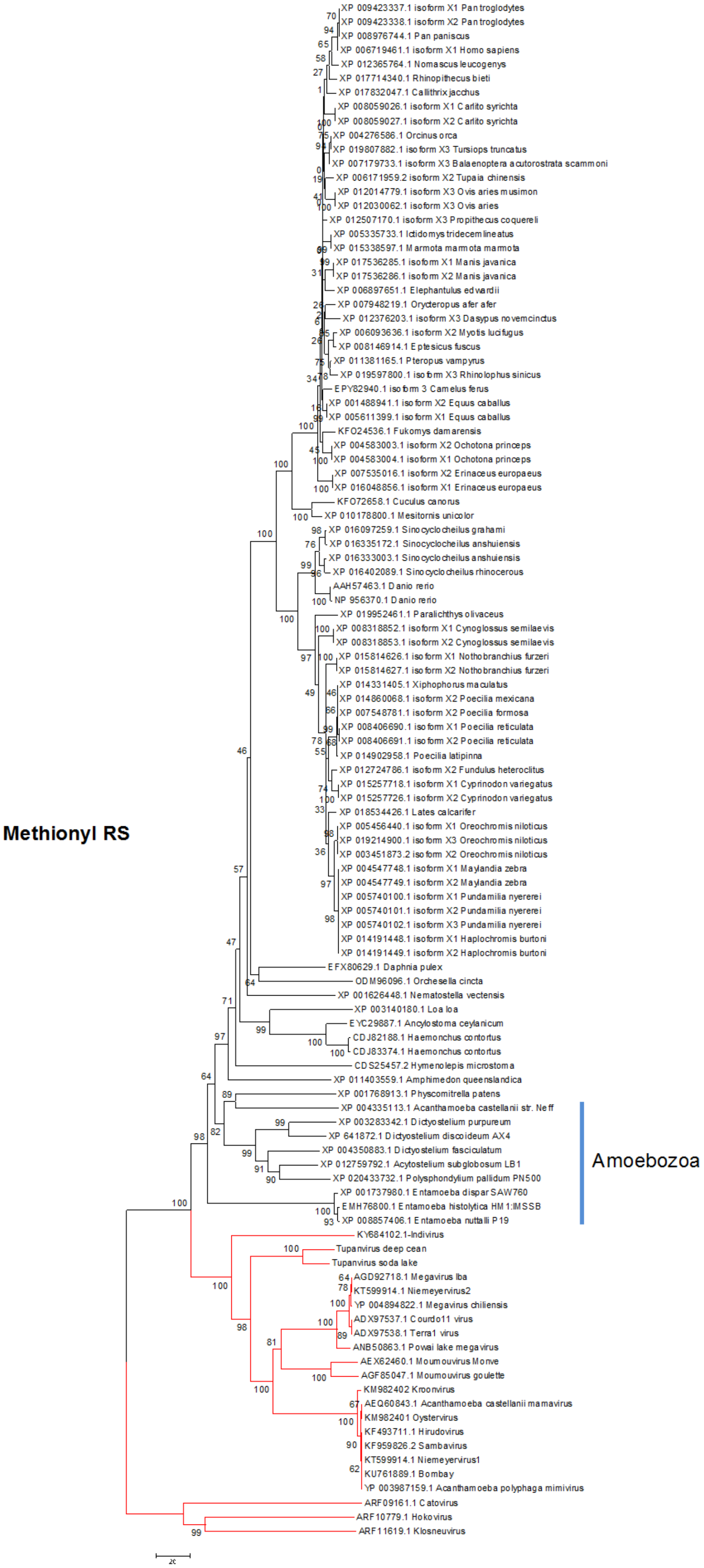
XP 009423337.1 isoform X1 Pan troglodytes
XP 009423338.1 isoform X2 Pan troglodytes
XP 008976744.1 Pan paniscus
XP 006719461.1 isoform X1 Homo sapiens
XP 012365764.1 Nomascus leucogenys
XP 017714340.1 Rhinopithecus bieti
XP 017832047.1 Callithrix jacchus
XP 008059026.1 isoform X1 Carlito syrichta
XP 008059027.1 isoform X2 Carlito syrichta
XP 004276586.1 Orcinus orca
XP 019807882.1 isoform X3 Tursiops truncatus
XP 007179733.1 isoform X3 Balaenoptera acutorostrata scammoni
XP 006171959.2 isoform X2 Tupaia chinensis
XP 012014779.1 isoform X3 Ovis aries musimon
XP 012030062.1 isoform X3 Ovis aries
XP 012507170.1 isoform X3 Propithecus coquereli
XP 005335733.1 Ictidomys tridecemlineatus
XP 015338597.1 Marmota marmota marmota
XP 017536285.1 isoform X1 Manis javanica
XP 017536286.1 isoform X2 Manis javanica
XP 006897651.1 Elephantulus edwardii
XP 007948219.1 Orycteropus afer afer
XP 012376203.1 isoform X3 Dasypus novemcinctus
XP 006093636.1 isoform X2 Myotis lucifugus
XP 008146914.1 Eptesicus fuscus
XP 011381165.1 Pteropus vampyrus
XP 019597800.1 isoform X3 Rhinolophus sinicus
EPY82940.1 isoform 3 Camelus ferus
XP 001488941.1 isoform X2 Equus caballus
XP 005611399.1 isoform X1 Equus caballus
KFO24536.1 Fukomys damarensis
XP 004583003.1 isoform X2 Ochotona princeps
XP 004583004.1 isoform X1 Ochotona princeps
XP 007535016.1 isoform X2 Erinaceus europaeus
XP 016048856.1 isoform X1 Erinaceus europaeus
KFO72658.1 Cuculus canorus
XP 010178800.1 Mesitornis unicolor
XP 016097259.1 Sinocyclocheilus grahami
XP 016335172.1 Sinocyclocheilus anshuiensis
XP 016333003.1 Sinocyclocheilus anshuiensis
XP 016402089.1 Sinocyclocheilus rhinocerous
AAH57463.1 Danio rerio
NP 956370.1 Danio rerio
XP 019952461.1 Paralichthys olivaceus
XP 008318852.1 isoform X1 Cynoglossus semilaevis
XP 008318853.1 isoform X2 Cynoglossus semilaevis
XP 015814626.1 isoform X1 Nothobranchius furzeri
XP 015814627.1 isoform X2 Nothobranchius furzeri
XP 014331405.1 Xiphophorus maculatus
XP 014860068.1 isoform X2 Poecilia mexicana
XP 007548781.1 isoform X2 Poecilia formosa
XP 008406690.1 isoform X1 Poecilia reticulata
XP 008406691.1 isoform X2 Poecilia reticulata
XP 014902958.1 Poecilia latipinna
XP 012724786.1 isoform X2 Fundulus heteroclitus
XP 015257718.1 isoform X1 Cyprinodon variegatus
XP 015257726.1 isoform X2 Cyprinodon variegatus
XP 018534426.1 Lates calcarifer
XP 005456440.1 isoform X1 Oreochromis niloticus
XP 019214900.1 isoform X3 Oreochromis niloticus
XP 003451873.2 isoform X2 Oreochromis niloticus
XP 004547748.1 isoform X1 Maylandia zebra
XP 004547749.1 isoform X2 Maylandia zebra
XP 005740100.1 isoform X1 Pundamilia nyererei
XP 005740101.1 isoform X2 Pundamilia nyererei
XP 005740102.1 isoform X3 Pundamilia nyererei
XP 014191448.1 isoform X1 Haplochromis burtoni
XP 014191449.1 isoform X2 Haplochromis burtoni
EFX80629.1 Daphnia pulex
ODM96096.1 Orchesella cincta
XP 001626448.1 Nematostella vectensis
XP 003140180.1 Loa loa
EYC29887.1 Ancylostoma ceylanicum
CDJ82188.1 Haemonchus contortus
CDJ83374.1 Haemonchus contortus
CDS25457.2 Hymenolepis microstoma
XP 011403559.1 Amphimedon queenslandica
XP 001768913.1 Physcomitrella patens
XP 004335113.1 Acanthamoeba castellanii str. Neff
XP 003283342.1 Dictyostelium purpureum
XP 641872.1 Dictyostelium discoideum AX4
XP 004350883.1 Dictyostelium fasciculatum
XP 012759792.1 Acytostelium subglobosum LB1
XP 020433732.1 Polysphondylium pallidum PN500
XP 001737980.1 Entamoeba dispar SAW760
EMH76800.1 Entamoeba histolytica HM1:IMSSB
XP 008857406.1 Entamoeba nuttalli P19
KY684102.1-Indivirus
Tupanvirus deep ocean
Tupanvirus soda lake
AGD92718.1 Megavirus lba
KT599914.1 Niemeyervirus2
YP 004894822.1 Megavirus chiliensis
ADX97537.1 Courdo11 virus
ADX97538.1 Terra1 virus
ANB50863.1 Powai lake megavirus
AEX62460.1 Moumouvirus Monve
AGF85047.1 Moumouvirus goulette
KM982402 Kroonvirus
AEQ60843.1 Acanthamoeba castellanii mamavirus
KM982401 Oystervirus
KF493711.1 Hirudovirus
KF959826.2 Sambavirus
KT599914.1 Niemeyervirus1
KU761889.1 Bombay
YP 003987159.1 Acanthamoeba polyphaga mimivirus
ARF09161.1 Catovirus
ARF10779.1 Hokovirus
ARF11619.1 Klosneuvirus
Methionyl RS
Amoebozoa

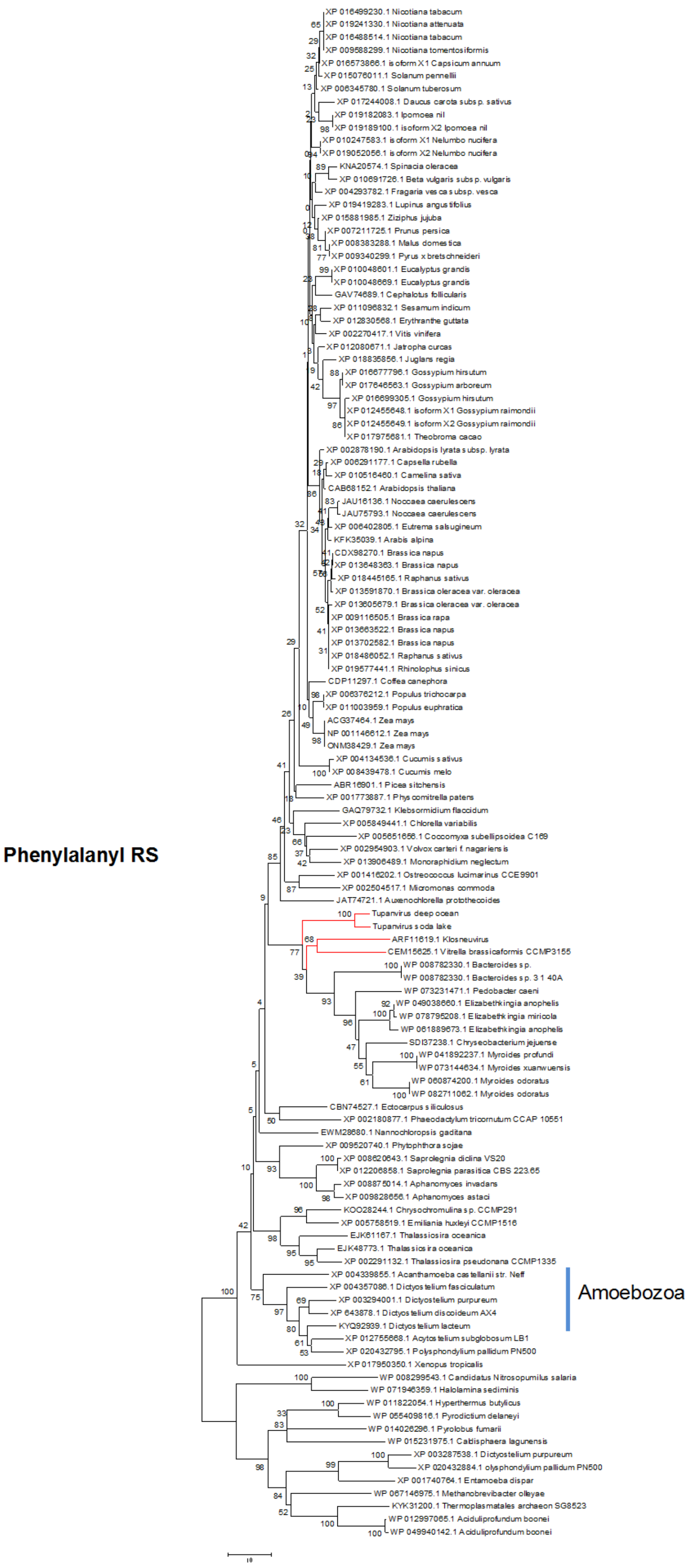
Phenylalanyl RS
XP 016499230.1 Nicotiana tabacum
XP 019241330.1 Nicotiana attenuata
XP 016488514.1 Nicotiana tabacum
XP 009588299.1 Nicotiana tomentosiformis
XP 016573866.1 isoform X1 Capsicum annuum
XP 015076011.1 Solanum pennellii
XP 006345780.1 Solanum tuberosum
XP 017244008.1 Daucus carota subsp. sativus
XP 019182083.1 Ipomoea nil
XP 019189100.1 isoform X2 Ipomoea nil
XP 010247583.1 isoform X1 Nelumbo nucifera
XP 019052056.1 isoform X2 Nelumbo nucifera
KNA20574.1 Spinacia oleracea
XP 010691726.1 Beta vulgaris subsp. vulgaris
XP 004293782.1 Fragaria vesca subsp. vesca
XP 019419283.1 Lupinus angustifolius
XP 015881985.1 Ziziphus jujuba
XP 007211725.1 Prunus persica
XP 008383288.1 Malus domestica
XP 009340299.1 Pyrus x bretschneideri
XP 010048601.1 Eucalyptus grandis
XP 010048669.1 Eucalyptus grandis
GAV74689.1 Cephalotus follicularis
XP 011096832.1 Sesamum indicum
XP 012830568.1 Erythranthe guttata
XP 002270417.1 Vitis vinifera
XP 012080671.1 Jatropha curcas
XP 018835856.1 Juglans regia
XP 016677796.1 Gossypium hirsutum
XP 017646563.1 Gossypium arboreum
XP 016699305.1 Gossypium hirsutum
XP 012455648.1 isoform X1 Gossypium raimondii
XP 012455649.1 isoform X2 Gossypium raimondii
XP 017975681.1 Theobroma cacao
XP 002878190.1 Arabidopsis lyrata subsp. lyrata
XP 006291177.1 Capsella rubella
XP 010516460.1 Camelina sativa
CAB68152.1 Arabidopsis thaliana
JAU16136.1 Noccaea caerulescens
JAU75793.1 Noccaea caerulescens
XP 006402805.1 Eutrema salsugineum
KFK35039.1 Arabis alpina
CDX98270.1 Brassica napus
XP 013648363.1 Brassica napus
XP 018445165.1 Raphanus sativus
XP 013591870.1 Brassica oleracea var. oleracea
XP 013605679.1 Brassica oleracea var. oleracea
XP 009116505.1 Brassica rapa
XP 013663522.1 Brassica napus
XP 013702582.1 Brassica napus
XP 018486052.1 Raphanus sativus
XP 019577441.1 Rhinolophus sinicus
CDP11297.1 Coffea canephora
XP 006376212.1 Populus trichocarpa
XP 011003959.1 Populus euphratica
ACG37464.1 Zea mays
NP 001146612.1 Zea mays
ONM38429.1 Zea mays
XP 004134536.1 Cucumis sativus
XP 008439478.1 Cucumis melo
ABR16901.1 Picea sitchensis
XP 001773887.1 Physcomitrella patens
GAQ79732.1 Klebsormidium flaccidum
XP 005849441.1 Chlorella variabilis
XP 005651656.1 Coccomyxa subellipsoidea C169
XP 002954903.1 Volvox carteri f. nagariensis
XP 013906489.1 Monoraphidium neglectum
XP 001416202.1 Ostreococcus lucimarinus CCE9901
XP 002504517.1 Micromonas commoda
JAT74721.1 Auxenochlorella protothecoides
Tupanvirus deep ocean
Tupanvirus soda lake
ARF11619.1 Klosneuvirus
CEM15625.1 Vitrella brassicaformis CCMP3155
WP 008782330.1 Bacteroides sp.
WP 008782330.1 Bacteroides sp. 3 1 40A
WP 073231471.1 Pedobacter caeni
WP 049038660.1 Elizabethkingia anophelis
WP 078795208.1 Elizabethkingia miricola
WP 061889673.1 Elizabethkingia anophelis
SDI37238.1 Chryseobacterium jejuense
WP 041892237.1 Myroides profundi
WP 073144634.1 Myroides xuanwuensis
WP 060874200.1 Myroides odoratus
WP 082711062.1 Myroides odoratus
CBN74527.1 Ectocarpus siliculosus
XP 002180877.1 Phaeodactylum tricornutum CCAP 10551
EWM28680.1 Nannochloropsis gaditana
XP 009520740.1 Phytophthora sojae
XP 008620643.1 Saprolegnia diclina VS20
XP 012206858.1 Saprolegnia parasitica CBS 223.65
XP 008875014.1 Aphanomyces invadans
XP 009828656.1 Aphanomyces astaci
KOO28244.1 Chrysochromulina sp. CCMP291
XP 005758519.1 Emiliania huxleyi CCMP1516
EJK61167.1 Thalassiosira oceanica
EJK48773.1 Thalassiosira oceanica
XP 002291132.1 Thalassiosira pseudonana CCMP1335
XP 004339855.1 Acanthamoeba castellanii str. Neff
XP 004357086.1 Dictyostelium fasciculatum
XP 003294001.1 Dictyostelium purpureum
XP 643878.1 Dictyostelium discoideum AX4
KYQ92939.1 Dictyostelium lacteum
XP 012755668.1 Acytostelium subglobosum LB1
XP 020432795.1 Polysphondylium pallidum PN500
Amoebozoa
XP 017950350.1 Xenopus tropicalis
WP 008299543.1 Candidatus Nitrosopumilus salaria
WP 071946359.1 Halolamina sediminis
WP 011822054.1 Hyperthermus butylicus
WP 055409816.1 Pyrodictium delaneyi
WP 014026296.1 Pyrolobus fumarii
WP 015231975.1 Caldisphaera lagunensis
XP 003287538.1 Dictyostelium purpureum
XP 020432884.1 olysphondylium pallidum PN500
XP 001740764.1 Entamoeba dispar
WP 067146975.1 Methanobrevibacter olleyae
KYK31200.1 Thermoplasmatales archaeon SG8523
WP 012997065.1 Aciduliprofundum boonei
WP 049940142.1 Aciduliprofundum boonei

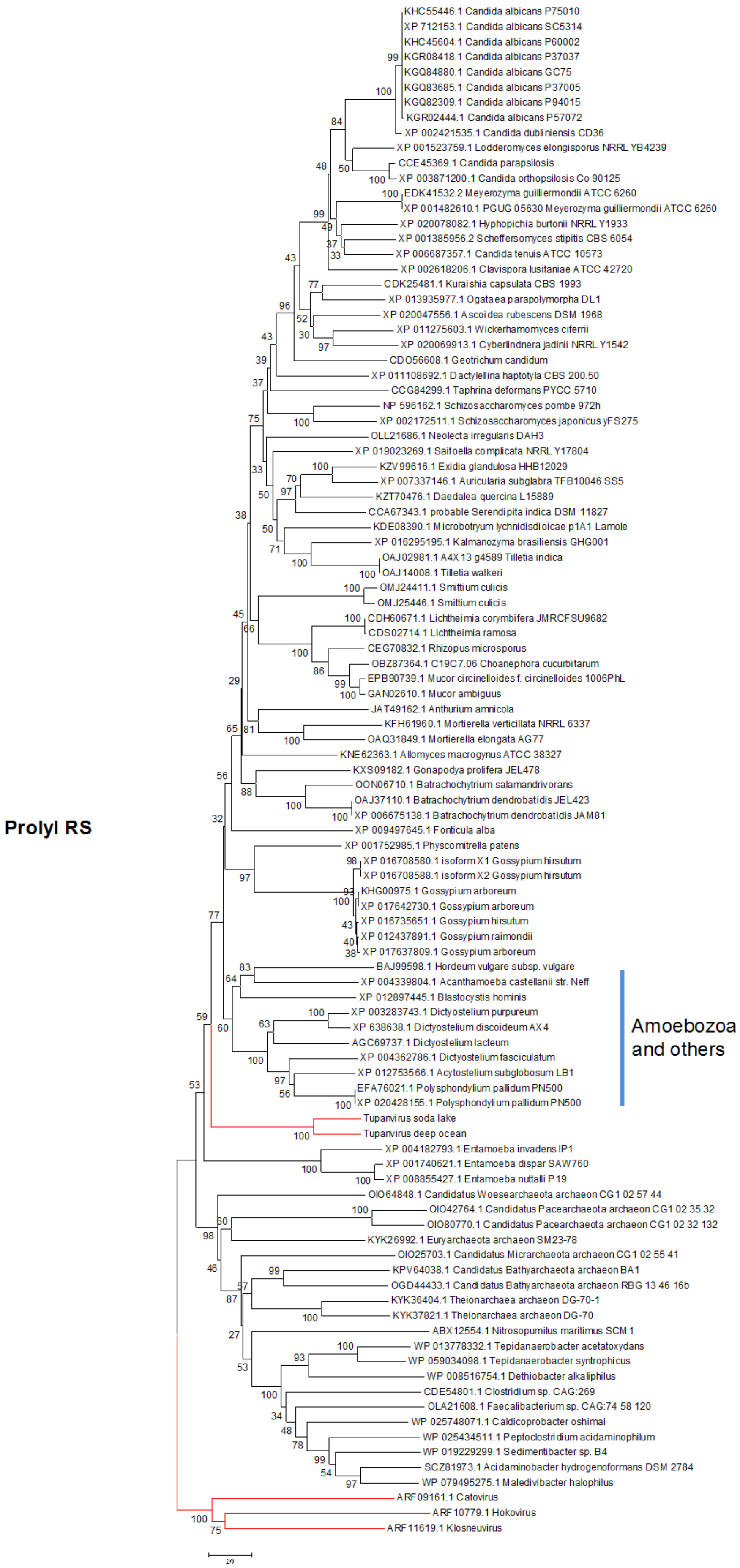
Prolyl RS
KHC55446.1 Candida albicans P75010
XP 712153.1 Candida albicans SC5314
KHC45604.1 Candida albicans P60002
KGR08418.1 Candida albicans P37037
KGQ84880.1 Candida albicans GC75
KGQ83685.1 Candida albicans P37005
KGQ82309.1 Candida albicans P94015
KGR02444.1 Candida albicans P57072
XP 002421535.1 Candida dubliniensis CD36
XP 001523759.1 Lodderomyces elongisporus NRRL YB4239
CCE45369.1 Candida parapsilosis
XP 003871200.1 Candida orthopsilosis Co 90125
EDK41532.2 Meyerozyma guilliermondii ATCC 6260
XP 001482610.1 PGUG 05630 Meyerozyma guilliermondii ATCC 6260
XP 020078082.1 Hyphopichia burtonii NRRL Y1933
XP 001385956.2 Scheffersomyces stipitis CBS 6054
XP 006687357.1 Candida tenuis ATCC 10573
XP 002618206.1 Clavispora lusitaniae ATCC 42720
CDK25481.1 Kuraishia capsulata CBS 1993
XP 013935977.1 Ogataea parapolymorpha DL1
XP 020047556.1 Ascoidea rubescens DSM 1968
XP 011275603.1 Wickerhamomyces ciferrii
XP 020069913.1 Cyberlindnera jadinii NRRL Y1542
CDO56608.1 Geotrichum candidum
XP 011108692.1 Dactylellina haptotyla CBS 200.50
CCG84299.1 Taphrina deformans PYCC 5710
NP 596162.1 Schizosaccharomyces pombe 972h
XP 002172511.1 Schizosaccharomyces japonicus yFS275
OLL21686.1 Neolecta irregularis DAH3
XP 019023269.1 Saitoella complicata NRRL Y17804
KZV99616.1 Exidia glandulosa HHB12029
XP 007337146.1 Auricularia subglabra TFB10046 SS5
KZT70476.1 Daedalea quercina L15889
CCA67343.1 probable Serendipita indica DSM 11827
KDE08390.1 Microbotryum lychnidisdioicae p1A1 Lamole
XP 016295195.1 Kalmanozyma brasiliensis GHG001
OAJ02981.1 A4X 13 g4589 Tilletia indica
OAJ14008.1 Tilletia walkeri
OMJ24411.1 Smittium culicis
OMJ25446.1 Smittium culicis
CDH60671.1 Lichtheimia corymbifera JMRCFSU9682
CDS02714.1 Lichtheimia ramosa
CEG70832.1 Rhizopus microsporus
OBZ87364.1 C19C7.06 Choanephora cucurbitarum
EPB90739.1 Mucor circinelloides f. circinelloides 1006PhL
GAN02610.1 Mucor ambiguus
JAT49162.1 Anthurium amnicola
KFH61960.1 Mortierella verticillata NRRL 6337
OAQ31849.1 Mortierella elongata AG77
KNE62363.1 Allomyces macrogynus ATCC 38327
KXS09182.1 Gonapodya prolifera JEL478
OON06710.1 Batrachochytrium salamandrivorans
OAJ37110.1 Batrachochytrium dendrobatidis JEL423
XP 006675138.1 Batrachochytrium dendrobatidis JAM81
XP 009497645.1 Fonticula alba
XP 001752985.1 Physcomitrella patens
XP 016708580.1 isoform X1 Gossypium hirsutum
XP 016708588.1 isoform X2 Gossypium hirsutum
KHG00975.1 Gossypium arboreum
XP 017642730.1 Gossypium arboreum
XP 016735651.1 Gossypium hirsutum
XP 012437891.1 Gossypium raimondii
XP 017637809.1 Gossypium arboreum
BAJ99598.1 Hordeum vulgare subsp. vulgare
XP 004339804.1 Acanthamoeba castellanii str. Neff
XP 012897445.1 Blastocystis hominis
XP 003283743.1 Dictyostelium purpureum
XP 638638.1 Dictyostelium discoideum AX4
AGC69737.1 Dictyostelium lacteum
XP 004362786.1 Dictyostelium fasciculatum
XP 012753566.1 Acytostelium subglobosum LB1
EFA76021.1 Polysphondylium pallidum PN500
XP 020428155.1 Polysphondylium pallidum PN500
Amoebozoa and others
Tupanvirus soda lake
Tupanvirus deep ocean
XP 004182793.1 Entamoeba invadens IP1
XP 001740621.1 Entamoeba dispar SAW760
XP 008855427.1 Entamoeba nuttalli P19
OIO64848.1 Candidatus Woesearchaeota archaeon CG1 02 57 44
OIO42764.1 Candidatus Pacearchaeota archaeon CG1 02 35 32
OIO80770.1 Candidatus Pacearchaeota archaeon CG1 02 32 132
KYK26992.1 Euryarchaeota archaeon SM23-78
OIO25703.1 Candidatus Micrarchaeota archaeon CG1 02 55 41
KPV64038.1 Candidatus Bathyarchaeota archaeon BA1
OGD44433.1 Candidatus Bathyarchaeota archaeon RBG 13 46 16b
KYK36404.1 Theionarchaea archaeon DG-70-1
KYK37821.1 Theionarchaea archaeon DG-70
ABX12554.1 Nitrosopumilus maritimus SCM1
WP 013778332.1 Tepidanaerobacter acetatoxydans
WP 059034098.1 Tepidanaerobacter syntrophicus
WP 008516754.1 Dethiobacter alkaliphilus
CDE54801.1 Clostridium sp. CAG:269
OLA21608.1 Faecalibacterium sp. CAG:74 58 120
WP 025748071.1 Caldicoprobacter oshimai
WP 025434511.1 Peptoclostridium acidaminophilum
WP 019229299.1 Sedimentibacter sp. B4
SCZ81973.1 Acidaminobacter hydrogenoformans DSM 2784
WP 079495275.1 Maledivibacter halophilus
ARF09161.1 Catovirus
ARF10779.1 Hokovirus
ARF11619.1 Klosneuvirus

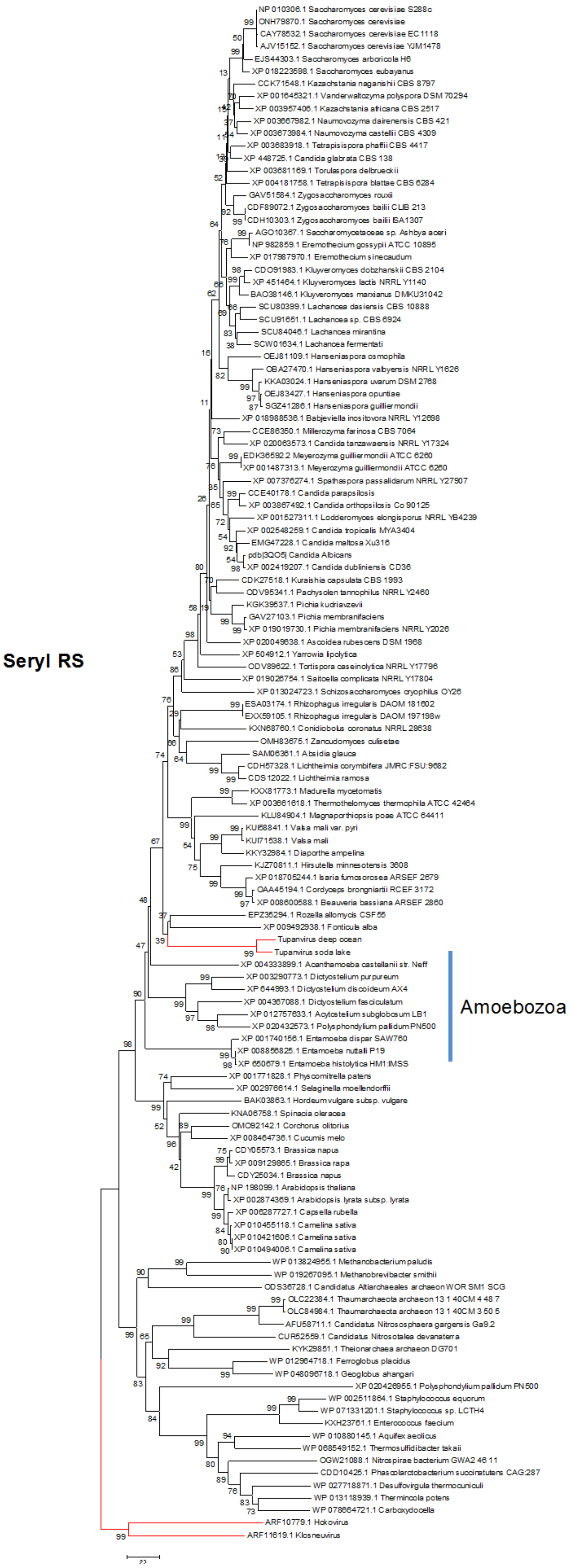
Seryl RS
Amoebozoa
NP 010306.1 Saccharomyces cerevisiae S288c
ONH79870.1 Saccharomyces cerevisiae
CAY78532.1 Saccharomyces cerevisiae EC1118
AJV15152.1 Saccharomyces cerevisiae YJM1478
EJS44303.1 Saccharomyces arboricola H6
XP 018223698.1 Saccharomyces eubayanus
CCK71548.1 Kazachstania naganishii CBS 8797
XP 001645321.1 Vanderwaltozyma polyspora DSM 70294
XP 003957406.1 Kazachstania africana CBS 2517
XP 003667982.1 Naumovozyma dairenensis CBS 421
XP 003673984.1 Naumovozyma castellii CBS 4309
XP 003683918.1 Tetrapisispora phaffii CBS 4417
XP 448725.1 Candida glabrata CBS 138
XP 003681169.1 Torulaspora delbrueckii
XP 004181758.1 Tetrapisispora blattae CBS 6284
GAV51584.1 Zygosaccharomyces rouxii
CDF89072.1 Zygosaccharomyces bailii CLIB 213
CDH10303.1 Zygosaccharomyces bailii ISA1307
AGO10367.1 Saccharomycetaceae sp. Ashbya aceri
NP 982859.1 Eremothecium gossypii ATCC 10895
XP 017987970.1 Eremothecium sinecaudum
CDO91983.1 Kluyveromyces dobzhanskii CBS 2104
XP 451464.1 Kluyveromyces lactis NRRL Y1140
BAO38146.1 Kluyveromyces marxianus DMKU31042
SCU80399.1 Lachancea dasiensis CBS 10888
SCU91651.1 Lachancea sp. CBS 6924
SCU84046.1 Lachancea mirantina
SCW01634.1 Lachancea fermentati
OEJ81109.1 Hanseniaspora osmophila
OBA27470.1 Hanseniaspora valbyensis NRRL Y1626
KKA03024.1 Hanseniaspora uvarum DSM 2768
OEJ83427.1 Hanseniaspora opuntiae
SGZ41286.1 Hanseniaspora guilliermondii
XP 018988536.1 Babjeviella inositovora NRRL Y12698
CCE86350.1 Millerozyma farinosa CBS 7064
XP 020063573.1 Candida tanzawaensis NRRL Y17324
EDK36592.2 Meyerozyma guilliermondii ATCC 6260
XP 001487313.1 Meyerozyma guilliermondii ATCC 6260
XP 007376274.1 Spathaspora passalidarum NRRL Y27907
CCE40178.1 Candida parapsilosis
XP 003867492.1 Candida orthopsilosis Co 90125
XP 001527311.1 Lodderomyces elongisporus NRRL YB4239
XP 002548259.1 Candida tropicalis MYA3404
EMG47228.1 Candida maltosa Xu316
pdb|3QO5| Candida Albicans
XP 002419207.1 Candida dubliniensis CD36
CDK27518.1 Kuraishia capsulata CBS 1993
ODV95341.1 Pachysolen tannophilus NRRL Y2460
KGK39537.1 Pichia kudriavzevii
GAV27103.1 Pichia membranifaciens
XP 019019730.1 Pichia membranifaciens NRRL Y2026
XP 020049638.1 Ascoidea rubescens DSM 1968
XP 504912.1 Yarrowia lipolytica
ODV89622.1 Tortispora caseinolytica NRRL Y17796
XP 019026754.1 Saitoella complicata NRRL Y17804
XP 013024723.1 Schizosaccharomyces cryophilus OY26
ESA03174.1 Rhizophagus irregularis DAOM 181602
EXX59105.1 Rhizophagus irregularis DAOM 197198w
KXN68760.1 Conidiobolus coronatus NRRL 28638
OMH83675.1 Zancudomyces culisetae
SAM06361.1 Absidia glauca
CDH57328.1 Lichtheimia corymbifera JMRC:FSU:9682
CDS12022.1 Lichtheimia ramosa
KXX81773.1 Madurella mycetomatis
XP 003661618.1 Thermothelomyces thermophila ATCC 42464
KLU84904.1 Magnaporthiopsis poae ATCC 64411
KUI58841.1 Valsa mali var. pyri
KUI71538.1 Valsa mali
KKY32984.1 Diaporthe ampelina
KJZ70811.1 Hirsutella minnesotensis 3608
XP 018705244.1 Isaria fumosorosea ARSEF 2679
OAA45194.1 Cordyceps brongniartii RCEF 3172
XP 008600588.1 Beauveria bassiana ARSEF 2860
EPZ35294.1 Rozella allomycis CSF55
XP 009492938.1 Fonticula alba
Tupanvirus deep ocean
Tupanvirus soda lake
XP 004333899.1 Acanthamoeba castellanii str. Neff
XP 003290773.1 Dictyostelium purpureum
XP 644993.1 Dictyostelium discoideum AX4
XP 004367088.1 Dictyostelium fasciculatum
XP 012757633.1 Acytostelium subglobosum LB1
XP 020432573.1 Polysphondylium pallidum PN500
XP 001740156.1 Entamoeba dispar SAW760
XP 008856825.1 Entamoeba nuttalli P19
XP 650679.1 Entamoeba histolytica HM1:IMSS
XP 001771828.1 Physcomitrella patens
XP 002976614.1 Selaginella moellendorffii
BAK03863.1 Hordeum vulgare subsp. vulgare
KNA06758.1 Spinacia oleracea
OMO92142.1 Corchorus olitorius
XP 008464736.1 Cucumis melo
CDY05573.1 Brassica napus
XP 009129865.1 Brassica rapa
CDY25034.1 Brassica napus
NP 198099.1 Arabidopsis thaliana
XP 002874369.1 Arabidopsis lyrata subsp. lyrata
XP 006287727.1 Capsella rubella
XP 010455118.1 Camelina sativa
XP 010421606.1 Camelina sativa
XP 010494006.1 Camelina sativa
WP 013824955.1 Methanobacterium paludis
WP 019267095.1 Methanobrevibacter smithii
ODS36728.1 Candidatus Altiarchaeales archaeon WOR SM1 SCG
OLC22384.1 Thaumarchaeota archaeon 13 1 40CM 4 48 7
OLC84984.1 Thaumarchaeota archaeon 13 1 40CM 3 50 5
AFU58711.1 Candidatus Nitrososphaera gargensis Ga9.2
CUR52559.1 Candidatus Nitrosotalea devanaterra
KYK29851.1 Theionarchaea archaeon DG701
WP 012964718.1 Ferroglobus placidus
WP 048096718.1 Geoglobus ahangari
XP 020426955.1 Polysphondylium pallidum PN500
WP 002511864.1 Staphylococcus equorum
WP 071331201.1 Staphylococcus sp. LCTH4
KXH23761.1 Enterococcus faecium
WP 010880145.1 Aquifex aeolicus
WP 068549152.1 Thermosulfidibacter takaii
OGW21088.1 Nitrospirae bacterium GWA2 46 11
CDD10425.1 Phascolarctobacterium succinatutens CAG:287
WP 027718871.1 Desulfovirgula thermocuniculi
WP 013118939.1 Thermincola potens
WP 078664721.1 Carboxydocella
ARF10779.1 Hokovirus
ARF11619.1 Klosneuvirus

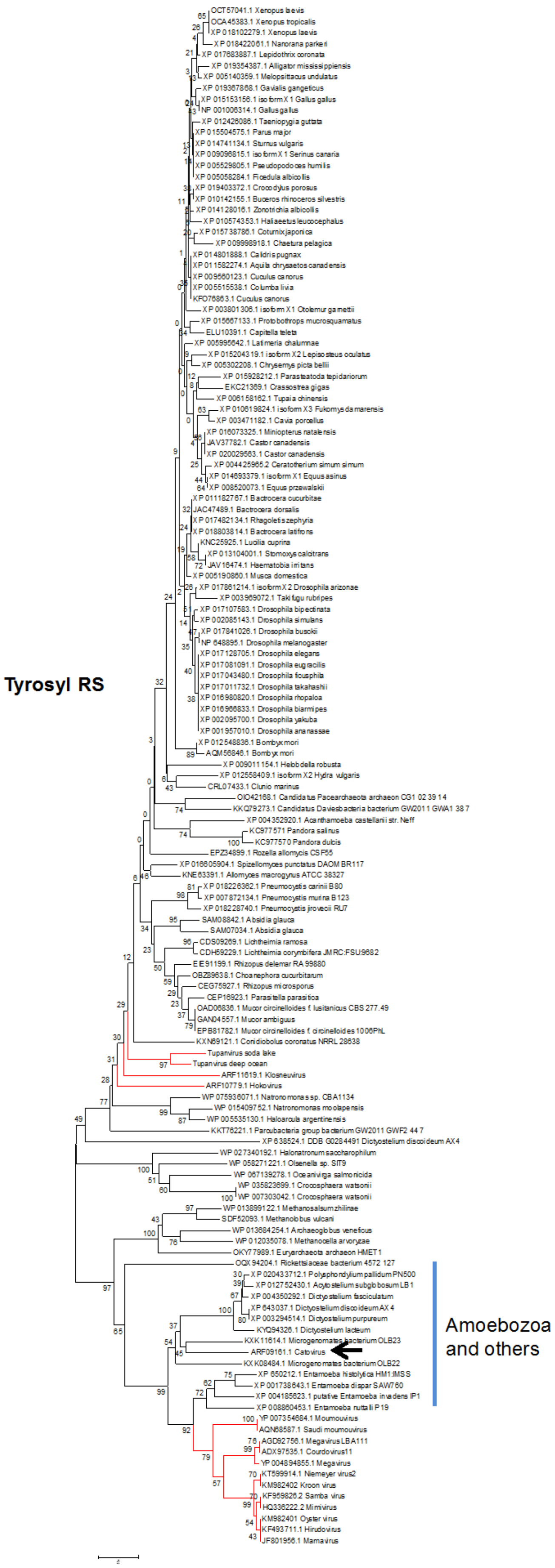
Tyrosyl RS
Amoebozoa and others

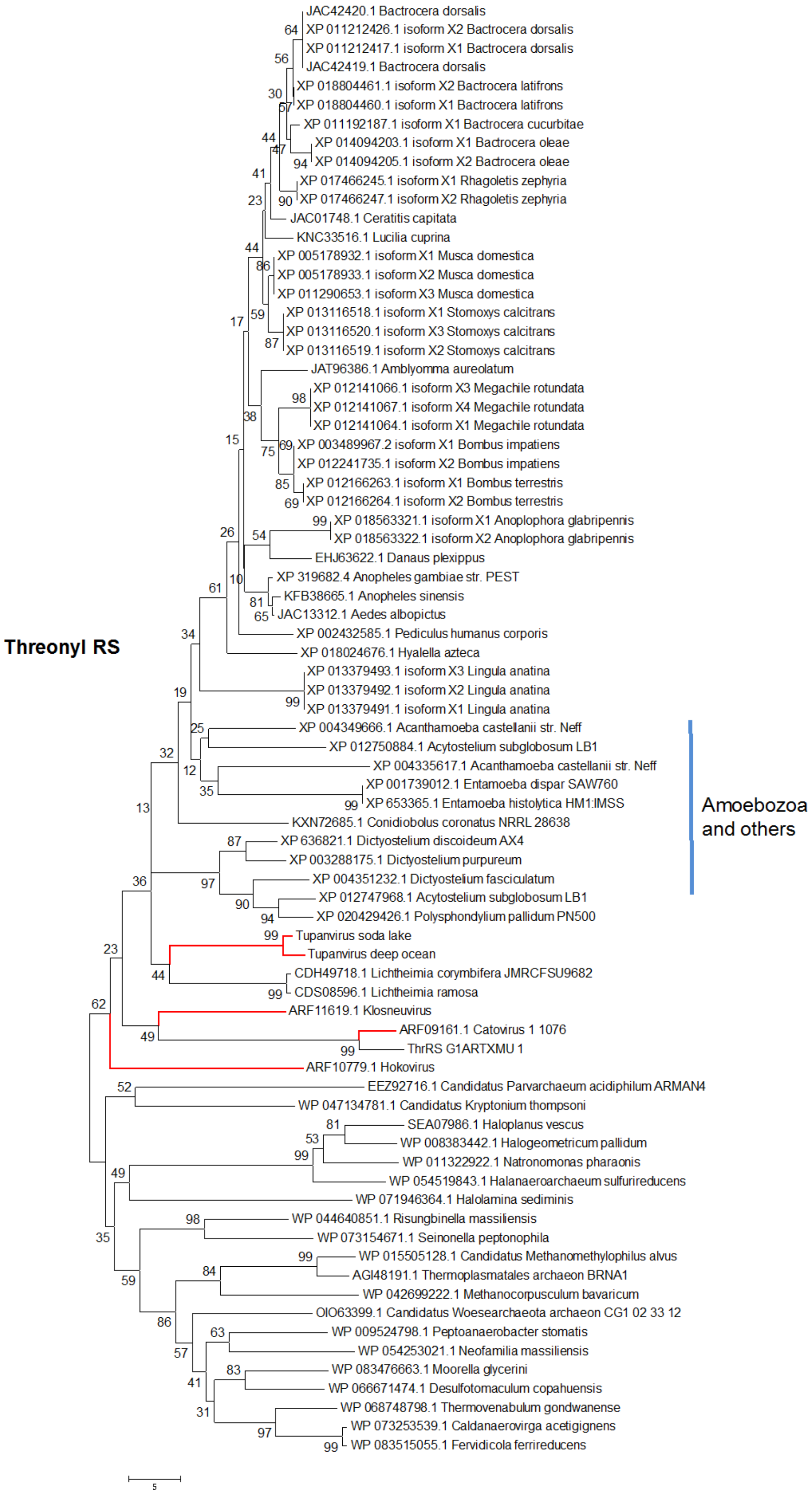
Threonyl RS
JAC42420.1 Bactrocera dorsalis
XP 011212426.1 isoform X2 Bactrocera dorsalis
XP 011212417.1 isoform X1 Bactrocera dorsalis
JAC42419.1 Bactrocera dorsalis
XP 018804461.1 isoform X2 Bactrocera latifrons
XP 018804460.1 isoform X1 Bactrocera latifrons
XP 011192187.1 isoform X1 Bactrocera cucurbitae
XP 014094203.1 isoform X1 Bactrocera oleae
XP 014094205.1 isoform X2 Bactrocera oleae
XP 017466245.1 isoform X1 Rhagoletis zephyria
XP 017466247.1 isoform X2 Rhagoletis zephyria
JAC01748.1 Ceratitis capitata
KNC33516.1 Lucilia cuprina
XP 005178932.1 isoform X1 Musca domestica
XP 005178933.1 isoform X2 Musca domestica
XP 011290653.1 isoform X3 Musca domestica
XP 013116518.1 isoform X1 Stomoxys calcitrans
XP 013116520.1 isoform X3 Stomoxys calcitrans
XP 013116519.1 isoform X2 Stomoxys calcitrans
JAT96386.1 Amblyomma aureolatum
XP 012141066.1 isoform X3 Megachile rotundata
XP 012141067.1 isoform X4 Megachile rotundata
XP 012141064.1 isoform X1 Megachile rotundata
XP 003489967.2 isoform X1 Bombus impatiens
XP 012241735.1 isoform X2 Bombus impatiens
XP 012166263.1 isoform X1 Bombus terrestris
XP 012166264.1 isoform X2 Bombus terrestris
XP 018563321.1 isoform X1 Anoplophora glabripennis
XP 018563322.1 isoform X2 Anoplophora glabripennis
EHJ63622.1 Danaus plexippus
XP 319682.4 Anopheles gambiae str. PEST
KFB38665.1 Anopheles sinensis
JAC13312.1 Aedes albopictus
XP 002432585.1 Pediculus humanus corporis
XP 018024676.1 Hyalella azteca
XP 013379493.1 isoform X3 Lingula anatina
XP 013379492.1 isoform X2 Lingula anatina
XP 013379491.1 isoform X1 Lingula anatina
XP 004349666.1 Acanthamoeba castellanii str. Neff
XP 012750884.1 Acytostelium subglobosum LB1
XP 004335617.1 Acanthamoeba castellanii str. Neff
XP 001739012.1 Entamoeba dispar SAW760
XP 653365.1 Entamoeba histolytica HM1:IMSS
KXN72685.1 Conidiobolus coronatus NRRL 28638
XP 636821.1 Dictyostelium discoideum AX4
XP 003288175.1 Dictyostelium purpureum
XP 004351232.1 Dictyostelium fasciculatum
XP 012747968.1 Acytostelium subglobosum LB1
XP 020429426.1 Polysphondylium pallidum PN500
Amoebozoa
and others
Tupanvirus soda lake
Tupanvirus deep ocean
CDH49718.1 Lichtheimia corymbifera JMRCFSU9682
CDS08596.1 Lichtheimia ramosa
ARF11619.1 Klosneuvirus
ARF09161.1 Catovirus 1 1076
ThrRS G1ARTXMU 1
ARF10779.1 Hokovirus
EEZ92716.1 Candidatus Parvarchaeum acidiphilum ARMAN4
WP 047134781.1 Candidatus Kryptonium thompsoni
SEA07986.1 Haloplanus vescus
WP 008383442.1 Halogeometricum pallidum
WP 011322922.1 Natronomonas pharaonis
WP 054519843.1 Halanaeroarchaeum sulfurireducens
WP 071946364.1 Halolamina sediminis
WP 044640851.1 Risungbinella massiliensis
WP 073154671.1 Seinonella peptonophila
WP 015505128.1 Candidatus Methanomethylophilus alvus
AGI48191.1 Thermoplasmatales archaeon BRNA1
WP 042699222.1 Methanocorpusculum bavaricum
OIO63399.1 Candidatus Woesearchaeota archaeon CG1 02 33 12
WP 009524798.1 Peptoanaerobacter stomatis
WP 054253021.1 Neofamilia massiliensis
WP 083476663.1 Moorella glycerini
WP 066671474.1 Desulfotomaculum copahuensis
WP 068748798.1 Thermovenabulum gondwanense
WP 073253539.1 Caldanaerovirga acetigignens
WP 083515055.1 Fervidicola ferrireducens
5

Tryptophanyl RS

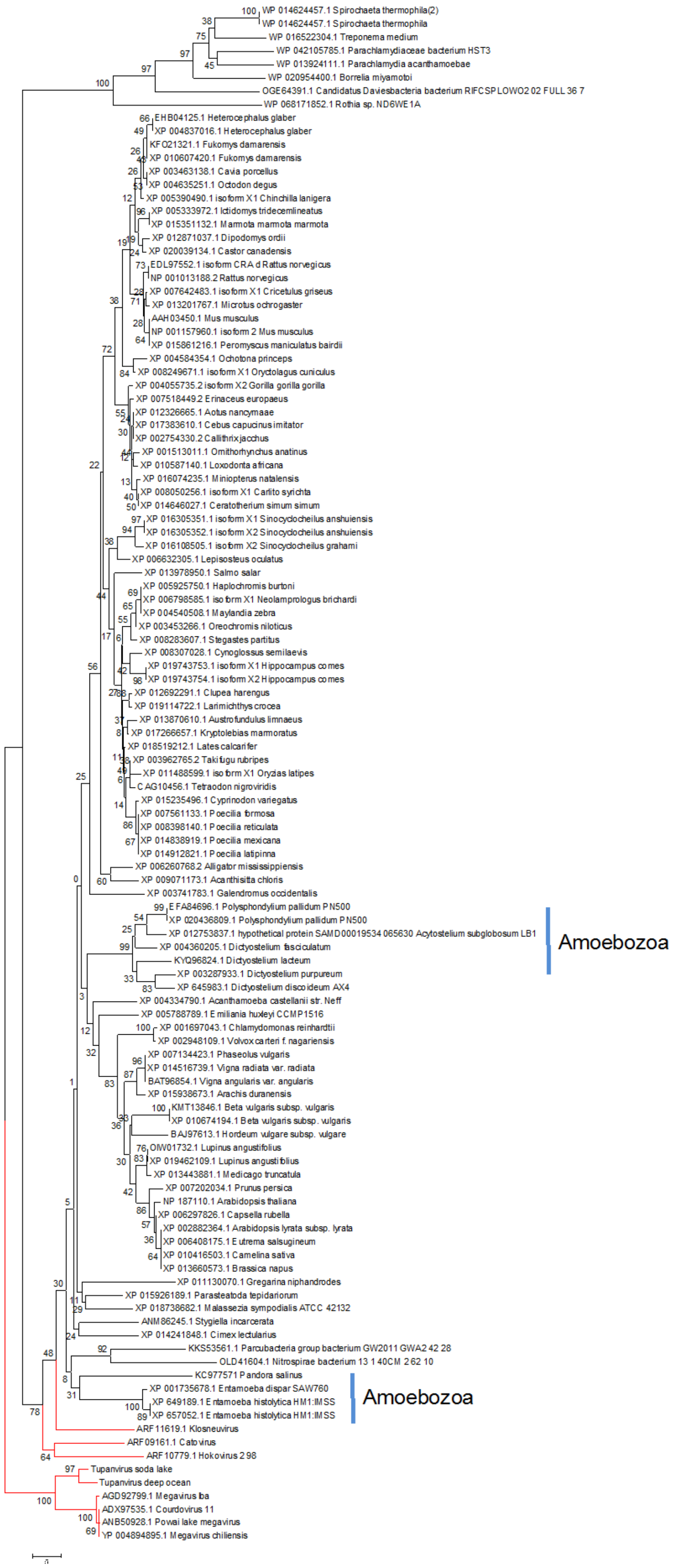

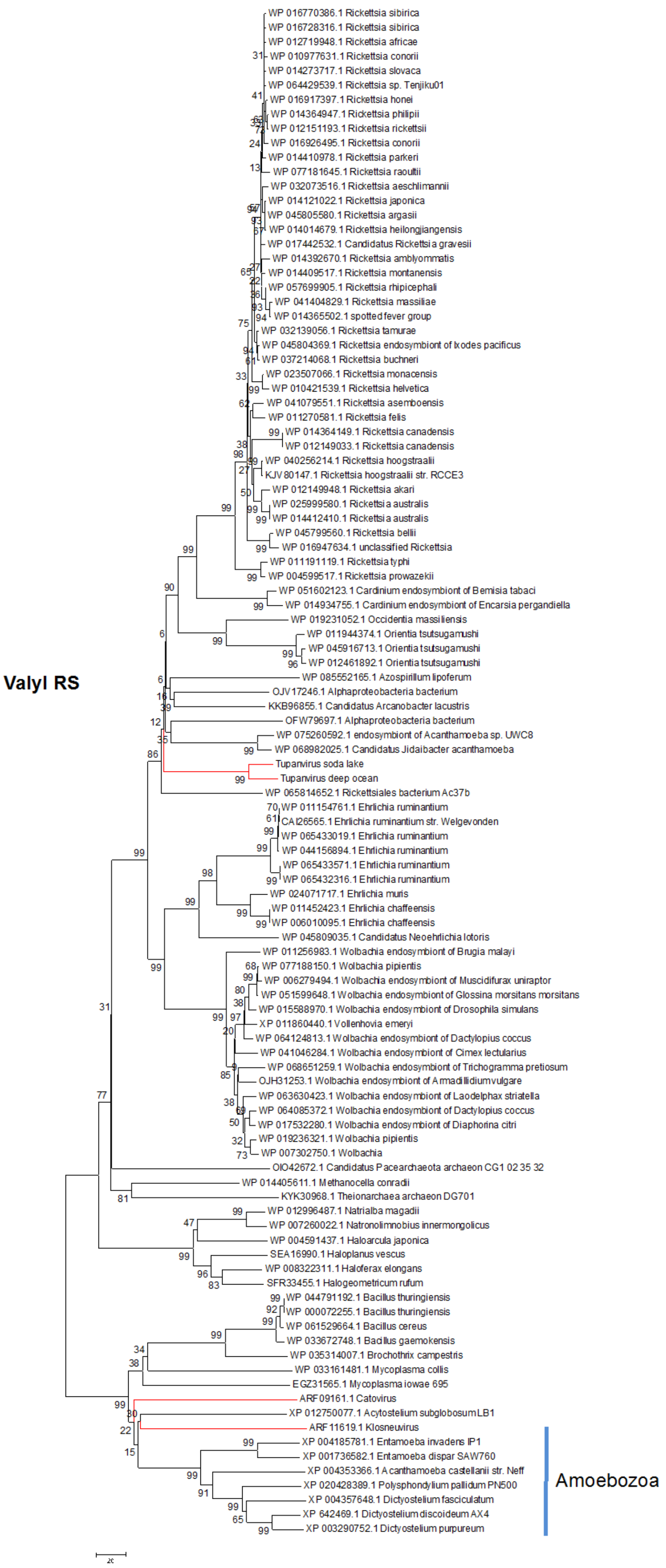
Valyl RS
WP 016770386.1 Rickettsia sibirica
WP 016728316.1 Rickettsia sibirica
WP 012719948.1 Rickettsia africae
WP 010977631.1 Rickettsia conorii
WP 014273717.1 Rickettsia slovaca
WP 064429539.1 Rickettsia sp. Tenjiku01
WP 016917397.1 Rickettsia honei
WP 014364947.1 Rickettsia philipii
WP 012151193.1 Rickettsia rickettsii
WP 016926495.1 Rickettsia conorii
WP 014410978.1 Rickettsia parkeri
WP 077181645.1 Rickettsia raoultii
WP 032073516.1 Rickettsia aeschlimannii
WP 014121022.1 Rickettsia japonica
WP 045805580.1 Rickettsia argasii
WP 014014679.1 Rickettsia heilongjiangensis
WP 017442532.1 Candidatus Rickettsia gravesii
WP 014392670.1 Rickettsia amblyommatis
WP 014409517.1 Rickettsia montanensis
WP 057699905.1 Rickettsia rhipicephali
WP 041404829.1 Rickettsia massiliae
WP 014365502.1 spotted fever group
WP 032139056.1 Rickettsia tamurae
WP 045804369.1 Rickettsia endosymbiont of Ixodes pacificus
WP 037214068.1 Rickettsia buchneri
WP 023507066.1 Rickettsia monacensis
WP 010421539.1 Rickettsia helvetica
WP 041079551.1 Rickettsia asembonensis
WP 011270581.1 Rickettsia felis
WP 014364149.1 Rickettsia canadensis
WP 012149033.1 Rickettsia canadensis
WP 040256214.1 Rickettsia hoogstraalii
KJV80147.1 Rickettsia hoogstraalii str. RCCE3
WP 012149948.1 Rickettsia akari
WP 025999580.1 Rickettsia australis
WP 014412410.1 Rickettsia australis
WP 045799560.1 Rickettsia bellii
WP 016947634.1 unclassified Rickettsia
WP 011191119.1 Rickettsia typhi
WP 004599517.1 Rickettsia prowazekii
WP 051602123.1 Cardinium endosymbiont of Bemisia tabaci
WP 014934755.1 Cardinium endosymbiont of Encarsia pergandiella
WP 019231052.1 Occidentia massiliensis
WP 011944374.1 Orientia tsutsugamushi
WP 045916713.1 Orientia tsutsugamushi
WP 012461892.1 Orientia tsutsugamushi
WP 085552165.1 Azospirillum lipoferum
OJV17246.1 Alphaproteobacteria bacterium
KKB96855.1 Candidatus Arcanobacter lacustris
OFW79697.1 Alphaproteobacteria bacterium
WP 075260592.1 endosymbiont of Acanthamoeba sp. UWC8
WP 068982025.1 Candidatus Jidaibacter acanthamoeba
Tupanvirus soda lake
Tupanvirus deep ocean
WP 065814652.1 Rickettsiales bacterium Ac37b
WP 011154761.1 Ehrlichia ruminantium
CAI26565.1 Ehrlichia ruminantium str. Welgevonden
WP 065433019.1 Ehrlichia ruminantium
WP 044156894.1 Ehrlichia ruminantium
WP 065433571.1 Ehrlichia ruminantium
WP 065432316.1 Ehrlichia ruminantium
WP 024071717.1 Ehrlichia muris
WP 011452423.1 Ehrlichia chaffeensis
WP 006010095.1 Ehrlichia chaffeensis
WP 045809035.1 Candidatus Neoehrlichia lotoris
WP 011256983.1 Wolbachia endosymbiont of Brugia malayi
WP 077188150.1 Wolbachia pipientis
WP 006279494.1 Wolbachia endosymbiont of Muscidifurax uniraptor
WP 051599648.1 Wolbachia endosymbiont of Glossina morsitans morsitans
WP 015588970.1 Wolbachia endosymbiont of Drosophila simulans
XP 011860440.1 Vollenhovia emeryi
WP 064124813.1 Wolbachia endosymbiont of Dactylopius coccus
WP 041046284.1 Wolbachia endosymbiont of Cimex lectularius
WP 068651259.1 Wolbachia endosymbiont of Trichogramma pretiosum
OJH31253.1 Wolbachia endosymbiont of Armadillidium vulgare
WP 063630423.1 Wolbachia endosymbiont of Laodelphax striatella
WP 064085372.1 Wolbachia endosymbiont of Dactylopius coccus
WP 017532280.1 Wolbachia endosymbiont of Diaphorina citri
WP 019236321.1 Wolbachia pipientis
WP 007302750.1 Wolbachia
OIO42672.1 Candidatus Pacearchaeota archaeon CG1 02 35 32
WP 014405611.1 Methanocella conradii
KYK30968.1 Theionarchaea archaeon DG701
WP 012996487.1 Natrialba magadii
WP 007260022.1 Natronolimnobius innermongolicus
WP 004591437.1 Haloarcula japonica
SEA16990.1 Haloplanus vescus
WP 008322311.1 Haloferax elongans
SFR33455.1 Halogeometricum rufum
WP 044791192.1 Bacillus thuringiensis
WP 000072255.1 Bacillus thuringiensis
WP 061529664.1 Bacillus cereus
WP 033672748.1 Bacillus gaemokensis
WP 035314007.1 Brochothrix campestris
WP 033161481.1 Mycoplasma collis
EGZ31565.1 Mycoplasma iowae 695
ARF09161.1 Catovirus
XP 012750077.1 Acytostelium subglobosum LB1
ARF11619.1 Klosneuvirus
XP 004185781.1 Entamoeba invadens IP1
XP 001736582.1 Entamoeba dispar SAW760
XP 004353366.1 Acanthamoeba castellanii str. Neff
XP 020428389.1 Polysphondylium pallidum PN500
XP 004357648.1 Dictyostelium fasciculatum
XP 642469.1 Dictyostelium discoideum AX4
XP 003290752.1 Dictyostelium purpureum
Amoebozoa

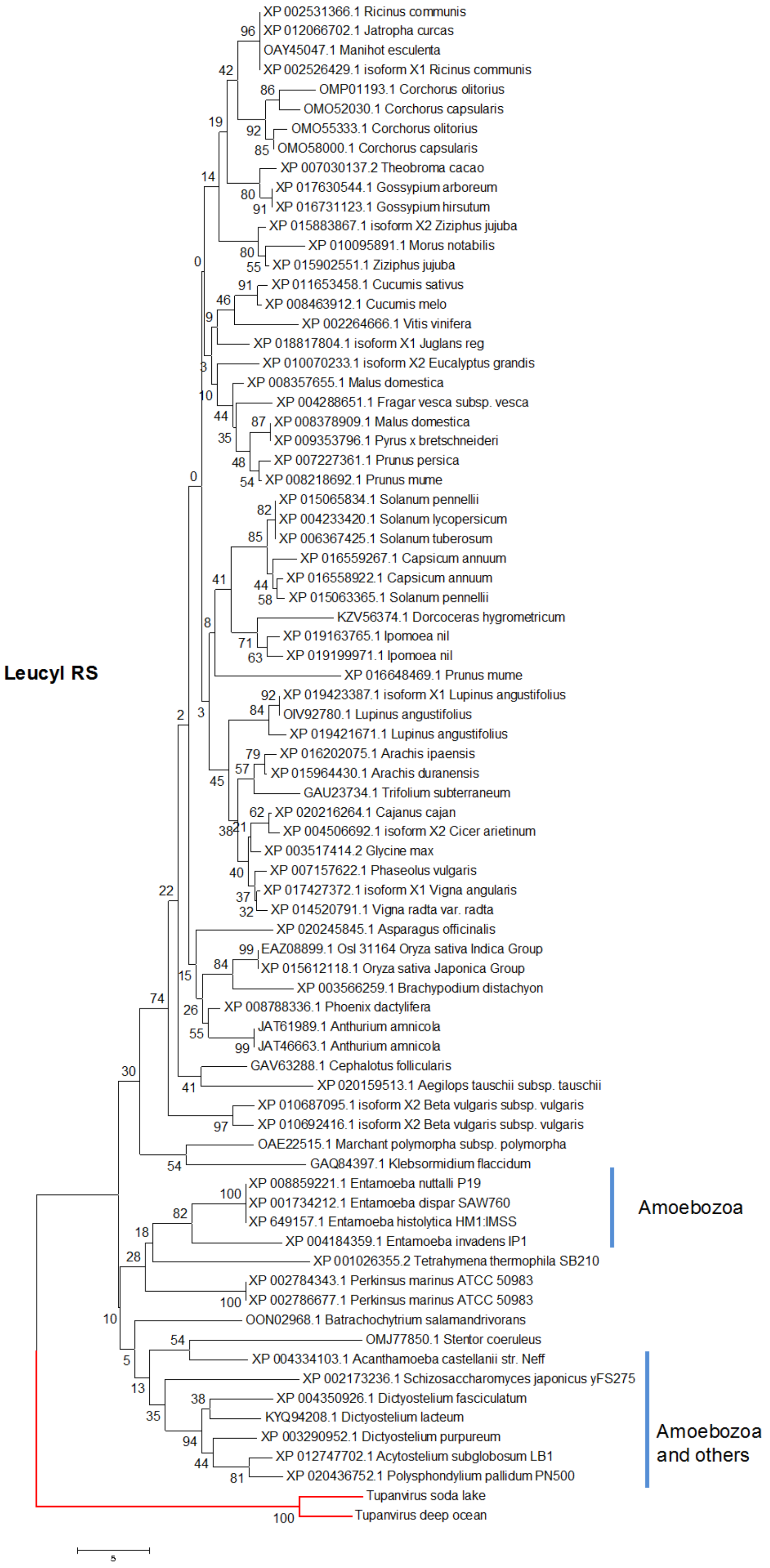
Leucyl RS
XP 002531366.1 Ricinus communis
XP 012066702.1 Jatropha curcas
OAY45047.1 Manihot esculenta
XP 002526429.1 isoform X1 Ricinus communis
OMP01193.1 Corchorus olitorius
OMO52030.1 Corchorus capsularis
OMO55333.1 Corchorus olitorius
OMO58000.1 Corchorus capsularis
XP 007030137.2 Theobroma cacao
XP 017630544.1 Gossypium arboreum
XP 016731123.1 Gossypium hirsutum
XP 015883867.1 isoform X2 Ziziphus jujuba
XP 010095891.1 Morus notabilis
XP 015902551.1 Ziziphus jujuba
XP 011653458.1 Cucumis sativus
XP 008463912.1 Cucumis melo
XP 002264666.1 Vitis vinifera
XP 018817804.1 isoform X1 Juglans reg
XP 010070233.1 isoform X2 Eucalyptus grandis
XP 008357655.1 Malus domestica
XP 004288651.1 Fragar vesca subsp. vesca
XP 008378909.1 Malus domestica
XP 009353796.1 Pyrus x bretschneideri
XP 007227361.1 Prunus persica
XP 008218692.1 Prunus mume
XP 015065834.1 Solanum pennellii
XP 004233420.1 Solanum lycopersicum
XP 006367425.1 Solanum tuberosum
XP 016559267.1 Capsicum annuum
XP 016558922.1 Capsicum annuum
XP 015063365.1 Solanum pennellii
KZV56374.1 Dorcoceras hygrometricum
XP 019163765.1 Ipomoea nil
XP 019199971.1 Ipomoea nil
XP 016648469.1 Prunus mume
XP 019423387.1 isoform X1 Lupinus angustifolius
OIV92780.1 Lupinus angustifolius
XP 019421671.1 Lupinus angustifolius
XP 016202075.1 Arachis ipaensis
XP 015964430.1 Arachis duranensis
GAU23734.1 Trifolium subterraneum
XP 020216264.1 Cajanus cajan
XP 004506692.1 isoform X2 Cicer arietinum
XP 003517414.2 Glycine max
XP 007157622.1 Phaseolus vulgaris
XP 017427372.1 isoform X1 Vigna angularis
XP 014520791.1 Vigna radta var. radta
XP 020245845.1 Asparagus officinalis
EAZ08899.1 OsI 31164 Oryza sativa Indica Group
XP 015612118.1 Oryza sativa Japonica Group
XP 003566259.1 Brachypodium distachyon
XP 008788336.1 Phoenix dactylifera
JAT61989.1 Anthurium amnicola
JAT46663.1 Anthurium amnicola
GAV63288.1 Cephalotus follicularis
XP 020159513.1 Aegilops tauschii subsp. tauschii
XP 010687095.1 isoform X2 Beta vulgaris subsp. vulgaris
XP 010692416.1 isoform X2 Beta vulgaris subsp. vulgaris
OAE22515.1 Marchantia polymorpha subsp. polymorpha
GAQ84397.1 Klebsormidium flaccidum
XP 008859221.1 Entamoeba nuttalli P19
XP 001734212.1 Entamoeba dispar SAW760
XP 649157.1 Entamoeba histolytica HM1:IMSS
XP 004184359.1 Entamoeba invadens IP1
XP 001026355.2 Tetrahymena thermophila SB210
XP 002784343.1 Perkinsus marinus ATCC 50983
XP 002786677.1 Perkinsus marinus ATCC 50983
OON02968.1 Batrachochytrium salamandrivorans
OMJ77850.1 Stentor coeruleus
XP 004334103.1 Acanthamoeba castellanii str. Neff
XP 002173236.1 Schizosaccharomyces japonicus yFS275
XP 004350926.1 Dictyostelium fasciculatum
KYQ94208.1 Dictyostelium lacteum
XP 003290952.1 Dictyostelium purpureum
XP 012747702.1 Acytostelium subglobosum LB1
XP 020436752.1 Polysphondylium pallidum PN500
Tupanvirus soda lake
Tupanvirus deep ocean
Amoebozoa
Amoebozoa and others
5

Supplementary Figure 5: The rhizome of the glutaminyl-tRNA synthetase of Tupanvirus soda lake. The sequence of the glutaminyl-tRNA synthetase was split into short fragments and blasted against the NR database. Best hit was selected and integrated in a circular gene data image. Taxonomic origins are colored in blue for eukaryote, green for bacteria and orange for viruses.

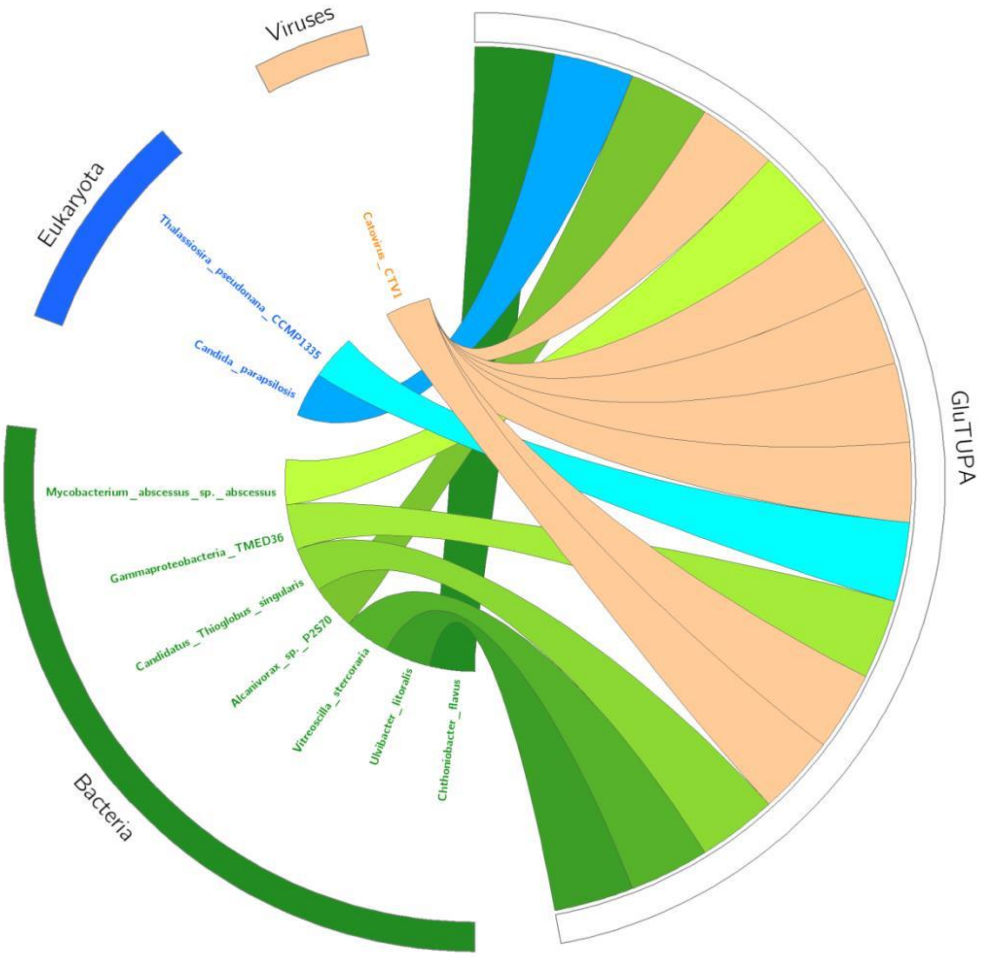

Supplementary Figure 6: Genomic environment and best-hit analyses of Tupanvirus soda lake 18S rRNA intronic regions. Genomic environment of copy 1 (a) and copy 2 (b). The 100 best hits for copies 1 (c) and 2 (d).

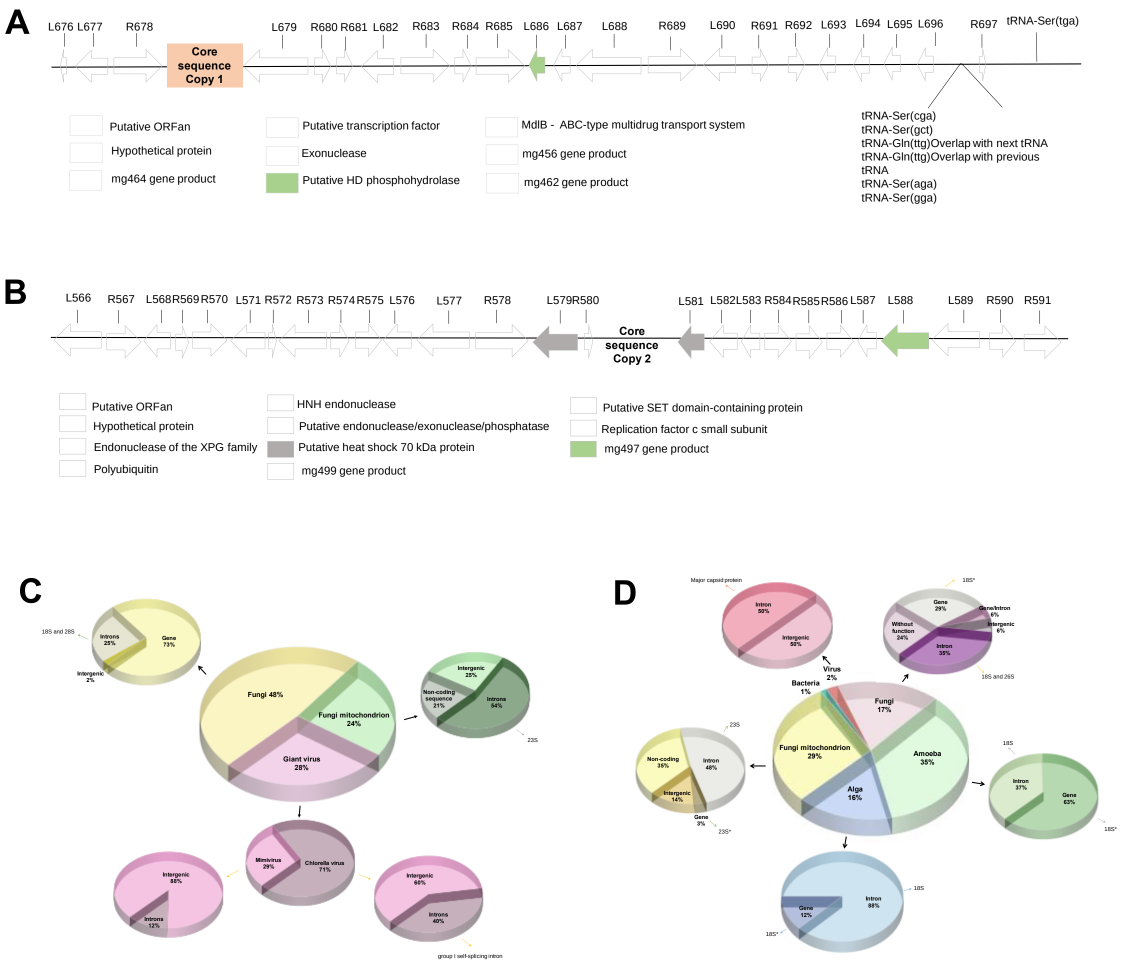

Supplementary Figure 7: Networks of translation-related category of genes of tupanviruses, Klosneuviruses, other mimiviruses and cell organisms.

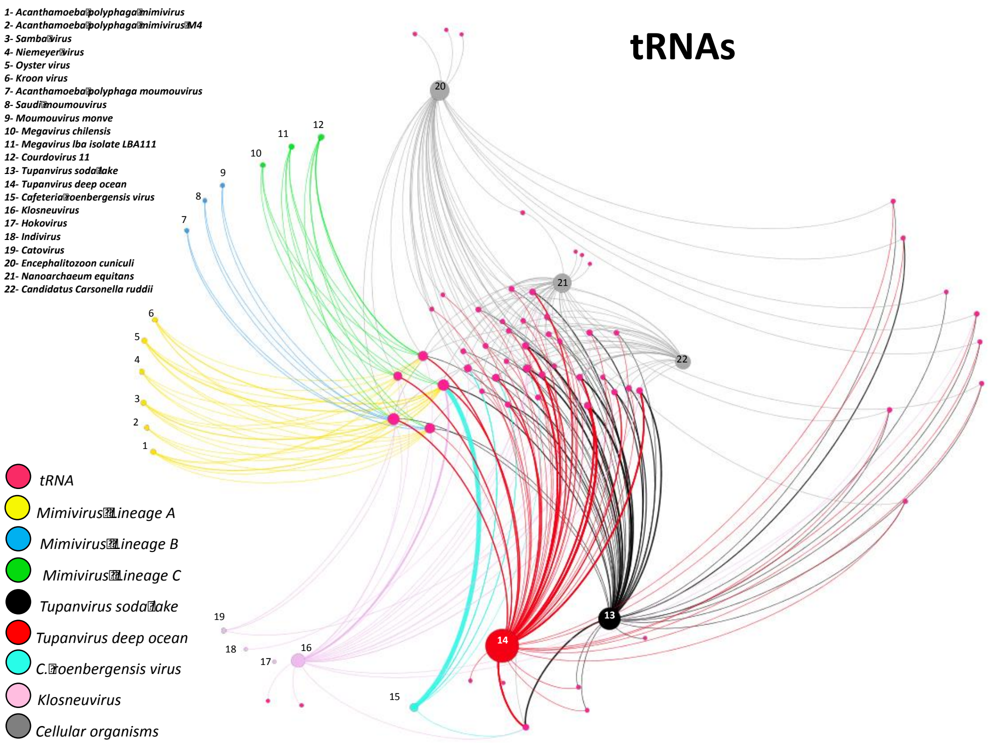

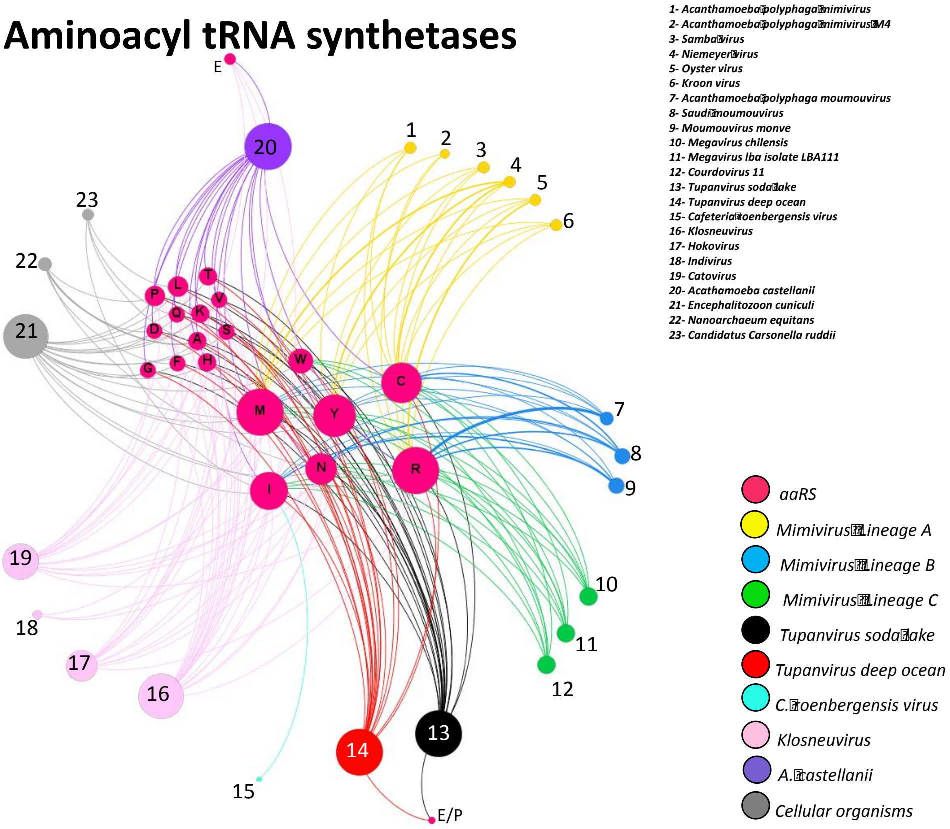

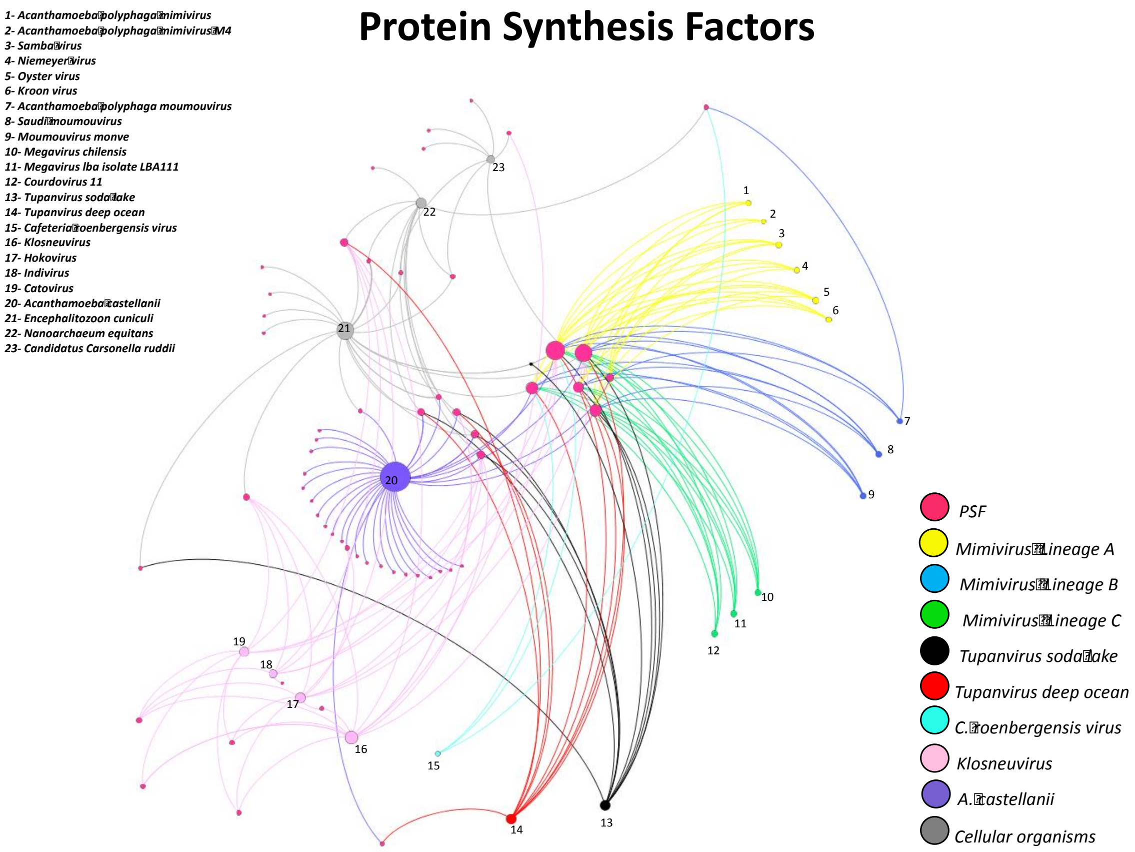
Protein Synthesis Factors
1- Acanthamoeba polyphaga mimivirus
2- Acanthamoeba polyphaga mimivirus M4
3- Samba virus
4- Niemeyer virus
5- Oyster virus
6- Kroon virus
7- Acanthamoeba polyphaga moumouvirus
8- Saudi moumouvirus
9- Moumouvirus monve
10- Megavirus chilensis
11- Megavirus lba isolate LBA111
12- Courdovirus 11
13- Tupanvirus soda lake
14- Tupanvirus deep ocean
15- Cafeteria roenbergensis virus
16- Klosneuvirus
17- Hokovirus
18- Indivirus
19- Catovirus
20- Acanthamoeba castellanii
21- Encephalitozoon cuniculi
22- Nanoarchaeum equitans
23- Candidatus Carsonella ruddii
PSF
Mimivirus Lineage A
Mimivirus Lineage B
Mimivirus Lineage C
Tupanvirus soda lake
Tupanvirus deep ocean
C. roenbergensis virus
Klosneuvirus
A. castellanii
Cellular organisms

Supplementary Figure 8: Analysis of Tupanvirus 18S rRNA intronic region (copies 1 or 2) expression in infected *Acanthamoeba castellanii* cells at 30 minutes and at 6 and 12 hours post-infection. Fluorescence in situ hybridization (FISH) (a and b) and qPCR (c – copy 1 and d – copy 2). In red, viral 18S rRNA intronic copies; in green *Acanthamoeba* 18S rRNA region (a and b). FISH analysis related to 24 hours post-infection; copy 1 is also shown in Figure 7a. Scale bar, 10 µm. Error bars (c and d), standard deviation. The experiments were performed 3 times independently, with two replicates each.

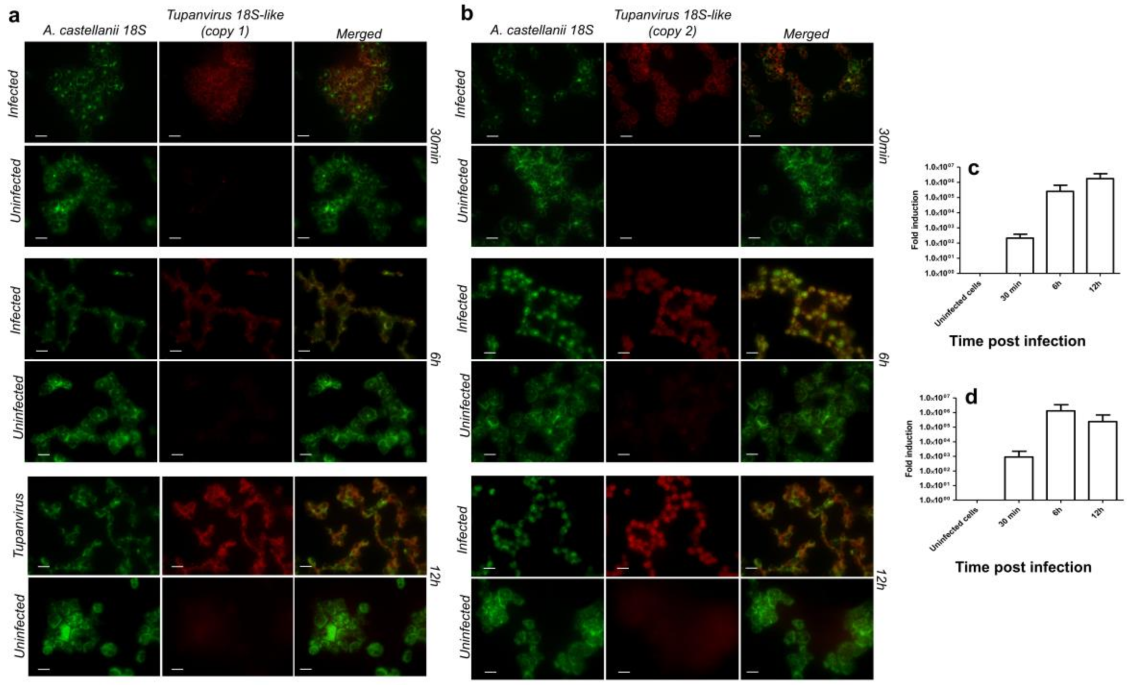

Supplementary Figure 9: Acidification of *Acanthamoeba castellanii* cytoplasm after Tupanvirus soda lake and mimivirus infection (9 h.p.i.). (a) Tupanvirus causes strong acidification of amoebal cytoplasm even in the presence of bafilomycin A. (b) Quantification of the acidification demonstrated in (a). (c-f) Even in the presence of chloroquine or bafilomycin A, the rRNA shutdown induced by Tupanvirus is not prevented (3 and 9 h.p.i.). (g-j) qPCR results related to the cells infected and treated as presented in (c-f). Scale bar, 5 µm. Error bars (b, g-j), standard deviation. Statistical analyses were performed on the data shown in (b) using a t-test based on control groups. **:p<0.01; ***:p<0.001. The experiments were performed 3 times independently, with two replicates each.

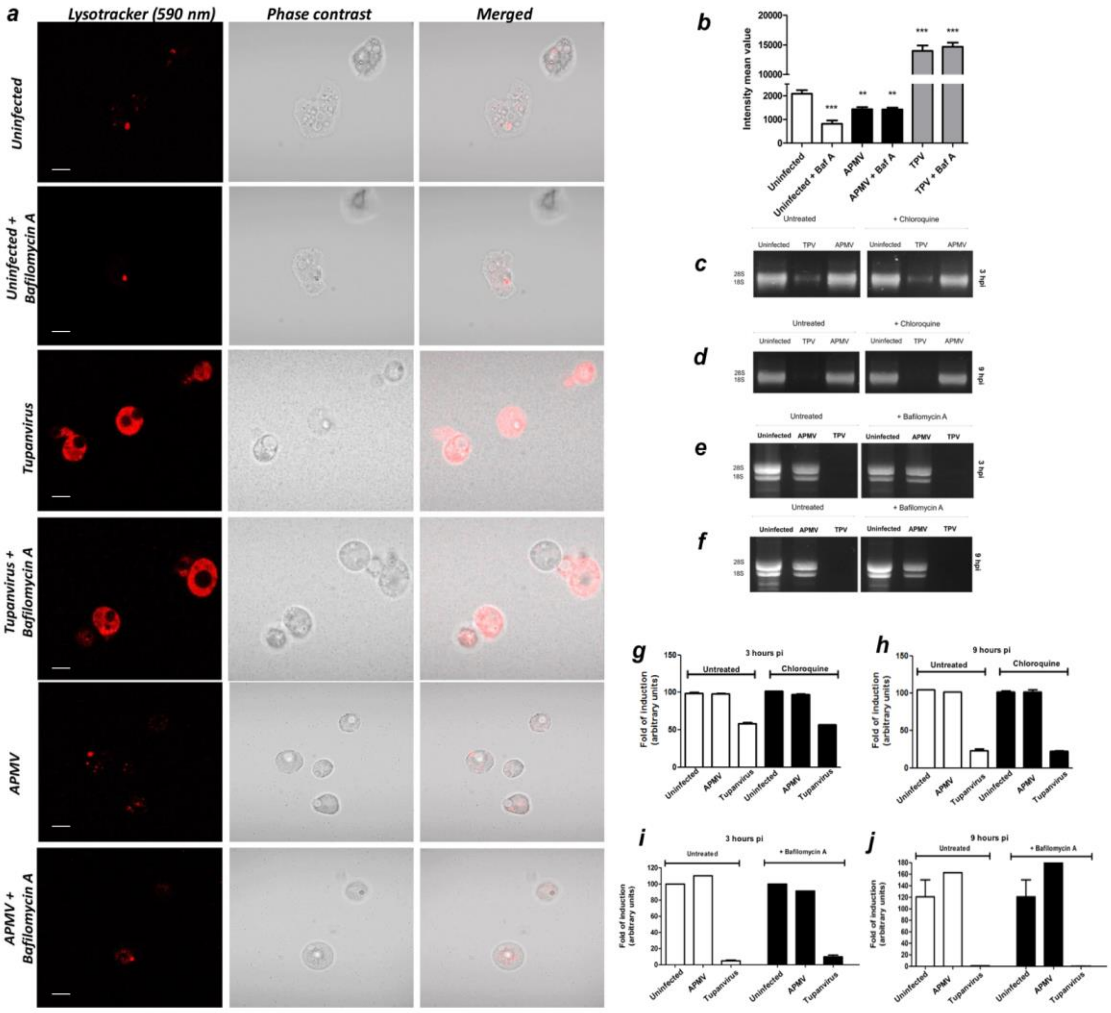

SUPPLEMENTARY TABLE

Supplementary Table 1: Permissiveness profile of Tupanvirus in different cells.

Cell	CPE	Viral titer increasing	Viral genome replication	Infection profile
Acanthamoeba castellanii	+	$3log_{10}$, after 24 hours	5 x $3log_{10}$, after 24 hours	productive
Acanthamoeba sp E4	+	$1log_{10}$, after 24 hours	6 x $3log_{10}$, after 24 hours	productive
Acanthamoeba sp. micheline	+	No	2 x $1log_{10}$, after 24 hours	abortive
Acanthamoeba polyphaga	+	$1log_{10}$, after 24 hours	4 x $1log_{10}$, after 24 hours	productive
Acanthamoeba royreba	+	No	2 x $1log_{10}$, after 24 hours	abortive
Acanthamoeba griffini	+	$1,5log_{10}$, after 24 hours	7 x $1,5log_{10}$, after 24 hours	productive
Vermamoeba vermiformis	+	$1log_{10}$, after 24 hours	6 x $1log_{10}$, after 24 hours	productive
Dyctiostelium discoideum	+	$1log_{10}$ after 48 hours	2 x $1log_{10}$ after 48 hours	productive
Willartia magna	+	$0,5log_{10}$ after 24 hours	$0,8log_{10}$ after 24 hours	productive
Tetrahymena hyperangularis	+	No	No	cytotoxic
THP-1	+	No	No	cytotoxic
RAW264.7	+	No	No	cytotoxic
Trichomonas tenax	-	No	No	resistant

CPE: cytopathic effect
