# Supplementary material for: Tailed giant Tupanvirus possesses the most complete translational apparatus of the known virosphere
Source: Nat Commun. 2018 Feb 27;9:749. doi: 10.1038/s41467-018-03168-1 (PMC5829246; doi:10.1038/s41467-018-03168-1)
Supplement: Supplementary file 3 — Description of Additional Supplementary Files [file 41467_2018_3168_MOESM3_ESM.pdf]

Description of Additional Supplementary Files

File Name: Supplementary Data 1
Description: Core genes and pangenome raw data related to tupanviruses and other mimiviruses. Viruses (names and GenBank Accession numbers) for which gene sets were available and used for the study of the pangenome of the family Mimiviridae were as follows: Acanthamoeba polyphaga mimivirus (NC_014649); Acanthamoeba polyphaga mamavirus (JF801956); Terra2 virus (NC_023639); Lentille virus (AFYC01000001-10); Hirudovirus (KF493731); Samba virus (KF959826); Niemeyer virus (KT599914); Kroon virus (KM982402); Mimivirus shirakomae (AP017645); Mimivirus kasaii (AP017644); Mimivirus Bombay (KU761889); Acanthamoeba polyphaga moumouvirus (NC_020104); Monve virus (JN885994-JN886001); Moumouvirus goulette (KC008572); Saudi moumouvirus (KY110734); Megavirus chilensis (NC_016072); Courdo7 virus (JN885990-JN885993); Terra1 virus (KF527229); Courdo 11 virus (JX975216); Powai lake megavirus isolate 1 virus (KU877344); LBA111 virus (NC_020232); Organic Lake phycodnavirus 1 (HQ704802); Organic Lake phycodnavirus 2 (HQ704803); Yellowstone lake mimivirus (NC_028104); Phaeocystis globosa virus 12T (HQ634147); Phaeocystis globosa virus 14T (HQ634144); Phaeocystis globosa virus 16T (NC_021312); Chrysochromulina ericina virus (NC_028094); Klosneuvirus (KY684108-KY684123); Indivirus (KY684085-KY684102); Catovirus (KY684083-KY684084); Hokovirus (KY684103-KY684106); Cafeteria roenbergensis virus (NC_014637); Tupanvirus soda lake (KY523104); and Tupanvirus deep ocean (MF405918).

File Name: Supplementary Data 2
Description: Proteomic dataset of Tupanvirus particles and comparison with mimivirus and Cafeteria roenbergensis virus particles proteomics. Data files were processed using ProteinLynx Global Server version 3.0.2. (Waters, Saint-Quentin En Yvelines, France). Processing parameters were 250 counts for the low energy threshold, 100 counts for the elevated energy threshold and 750 counts for the intensity threshold. For each replicate, data files of the 7 fractions individually processed were merged in only one file for analysis. For peptide identification, data were searched against an in house annotated fasta files of Tupanvirus containing 1268 sequences. The enzyme specificity was trypsin allowing 1 missed cleavage, carbamidomethyl of cysteine (fixed), oxidation of methionine (variable), carbamyl of lysine and N-terminal (variable). Search tolerance parameters were as follows: peptide and fragment tolerance, 15 ppm, FDR < 4%. Minimum Ion matching requirements were three fragments per peptide, seven fragments per protein, and two peptides per protein. Proteins identifications for the 4 replicates was merge without filter. An average of the number of peptides matched was calculated. The identifications with this average of number of peptides lower than 2 and found only in one replicates were excluded. Finally, an average of the PLGS scores and an average of the amounts were calculated to classify proteins.

File Name: Supplementary Data 3
Description: Detailed list of Tupanvirus soda lake translation-related factors. We present in this file the best-hit analyses for each translation-related factor.

File Name: Supplementary Data 4
Description: Detailed list of Tupanvirus deep ocean translation-related factors. We present in this file the best-hit analyses for each translation-related factor.

File Name: Supplementary Movie 1
Description: Tomographic reconstruction of Tupanvirus factory in A. castellanii at 18 h post-infection. The acceleration voltage was 200 keV, the magnification was set to 6,500 and the image pixel size was 1.64 nm. The tilt series ranged from -50° to +50° in 1° steps. The dimensions of the 3D reconstruction from the corresponding tilt series were 6.5 x 5.4 µm in XY and 268.96 nm in Z.

Tupanvirus at distinct locations in the cell and different maturation stages can be observed. Scale bar is 500 nm.

File Name: Supplementary Movie 2
Description: Zoom-in tomogram from Movie 1 and segmentation of the tail-capsid junction over a single Tupanvirus. Left: tomogram; Right: binning 2/smoothing 1 iso-surface. Movie finishing on structurally separated tail and capsid, with fibres joining the two entities. Slices shown are 3.7 nm apart in Z over a 40.7-nm vertical range. Scale bar is 110 nm.
